# Biochar-Driven Activity Enhancement of Co_3_O_4_ Catalyst for 4-Nitrophenol Reduction: RSM-CCD Optimization, Kinetic Insights, and Recyclability

**DOI:** 10.3390/molecules31173119

**Published:** 2026-09-06

**Authors:** Layla El Moussaoui, Majda Ben Ali, Abdellah Benzaouak, Oumaima Sadik, Mohammed Dahhou, Abdelekbir Bellaouchou, Adnane El Hamidi, Abdelouahed Lokmane

**Affiliations:** 1 Laboratory of Materials, Nanotechnologies and Environment, Center of Materials Science, Faculty of Sciences, Mohammed V University in Rabat, B.P.1014 RP, Rabat 10100, Morocco; layla_elmoussaoui@um5.ac.ma (L.E.M.); majda-benali@um5r.ac.ma (M.B.A.); oumaimasadik380@gmail.com (O.S.); m.dahhou@um5r.ac.ma (M.D.); a.bellaouchou@um5r.ac.ma (A.B.); a.elhamidi@um5r.ac.ma (A.E.H.); 2 Laboratory of Spectroscopy, Molecular Modeling, Materials, Nanomaterials, Water and Environment, Environmental Materials Team, ENSAM, Mohammed V University in Rabat, B.P. 6207, Rabat 10100, Morocco; a.benzaouak@um5r.ac.ma; 3 Laboratoire de Sécurité des Procédés Chimiques LSPC EA-4704, INSA Rouen Normandie, University Rouen Normandie, UNIROUEN, 76000 Rouen, France

**Keywords:** urban sludge, Co_3_O_4_ nanoparticles, Co_3_O_4_/biochar composites, reusability and stability, catalytic reduction, 4-NP

## Abstract

To decrease the environmental and health consequences associated with pollution, several cobalt oxides-decorated biochar substrates have been developed for effective catalytic reduction of 4-NP. The porous biochar was produced by pyrolysis of sewage sludge and acid demineralization, and subsequently, Co_3_O_4_ was loaded with varying amounts to synthesize Co_3_O_4_/biochar nanocomposites. The characterization of the materials included FTIR, XRD, SEM/EDX, TEM, BET, CHNS elemental analysis, TGA/DTA, and pH_pzc_ analysis. The catalytic activity of synthesized composites was monitored by UV-Vis spectroscopy to measure the transformation of a priority toxic pollutant, 4-NP, to a desirable pharmaceutical intermediate, 4-AP, by NaBH4, considering it a reducing agent. The influence of corresponding parameters such as catalyst dosage, NaBH_4_ concentration, and Co_3_O_4_ content in the composite on the reduction time of 4-NP was modelled using central composite design (CCD) of RSM. The optimal conditions for the catalytic reduction of 0.3 mM 4-NP were found to be a catalyst dosage of 5 mg, a NaBH_4_ concentration of 26 mM, and a Co_3_O_4_ loading of 13%, resulting in complete reduction within 5 min. The optimized catalyst displayed a high porosity, well dispersion of Co_3_O_4_ nanoparticles, good catalytic stability, and cost-effectiveness, revealing new insights into application of cobalt oxide and sewage sludge for pollutant reduction.

## 1. Introduction

4-nitrophenol (4-NP) is a popular organic contaminant in wastewater due to its industrial use in pesticides, dyes, drugs, and other chemicals. The 4-NP is chemically stable and water-soluble, which results in a gradual dissipation of aquatic environment pollution over a long time [1,2,3]. While conventional treatment processes can remove some organic pollutants, 4-NP remains a significant challenge [4]. Toxicity testing indicated that 4-NP is a toxic substance for organisms, and even at lower concentrations, it can affect human health, causing neurotoxicity, endocrine disorders, and kidney damage. 4-NP is classified as a priority pollutant in both the USA and the EU, and its removal from natural waters is a must for the environment and health [5].

Among the various strategies developed for the elimination of such recalcitrant pollutants, catalytic reduction has emerged as an efficient and promising approach.

Metal oxides are widely used in catalysis of 4-NP reduction in the presence of NaBH_4_ as a reducing agent [6,7]. In this context, cobalt oxide (Co_3_O_4_) is mentioned as an excellent catalyst due to its high activity, chemical and thermal stability, and favorable electronic conductivity [8,9]. Its spinel structure facilitates the interconversion between the Co^2+^ and Co^3+^ oxidation states, which gives it very high reactivity in pollutant degradation processes [10,11]. However, the catalytic performance of cobalt oxide is limited by poor catalyst recovery and particle agglomeration, which reduces the number of exposed active sites and, therefore, lowers its catalytic activity. The catalytic performance of Co_3_O_4_ to reduce 4NP has been investigated in many studies by doping [12,13] or supporting on a porous surface [14,15] to facilitate adsorption process and electron transfer, hence enhancing catalytic activity.

Recent years have seen an increasing focus on using waste-derived biomass to produce biochar because this practice supports both sustainable development and circular economy principles.

Urban sludge is a waste generated from the treatment of water previously used for both domestic and industrial activities [16,17]. Besides its environmental impact, the continued generation of urban sludge presents certain compositional difficulties. Urban sludge usually consists of biodegradable organic matter, inorganic compounds, nutrients (like nitrogen and phosphorus), as well as some heavy metals (e.g., lead, cadmium, and mercury) and pathogenic agents consisting of bacteria, viruses, and parasites [18,19,20]. Thus, urban sludge must be properly treated and disposed of to lessen its environmental and health impacts [21,22]. Among the available methods, pyrolysis serves to turn sewage sludge to biochar as a carbon-rich material by thermal decomposition under limited oxygen supply. The high specific surface area and good chemical and thermal stabilities of biochar make it suitable for use in a range of applications in the environmental sector, such as soil amendment, contaminant adsorption, and catalysis [23,24,25].

Therefore, waste sewage sludge could be considered as a potential source of carbonaceous materials instead of a source of pollution [26,27,28].

Incorporating Co_3_O_4_ into biochar improves the catalytic performance of pristine Co_3_O_4_. Due to the porous nature of biochar and its high surface area, there are many places where Co_3_O_4_ nanoparticles can be anchored; this facilitates even distribution and prevents agglomeration of nanoparticles. The total number of active sites available is maximized, and catalytic activity is retained. Also, the biochar presents a solid support to the recovery of the composite material after the reduction reaction and mitigates the typical separation challenges associated with dispersed oxide nanoparticles in aqueous systems [29].

The surface structure of biochar allows for increasing its adsorptive properties, promoting better contact between reactants, and thus improving the catalytic activity of 4-nitrophenol reduction in the presence of sodium borohydride [30].

Several studies have reported the effectiveness of metal oxide–biochar supported catalysts as efficient and recyclable systems for the reduction of 4-nitrophenol. In particular, the work of Lisette A. Ramirez, Mariana Dennehy, and Mariana Alvarez demonstrated that such hybrid materials exhibit remarkable catalytic performance due to the synergistic interaction between the metal oxide active phase and the biochar support. It is worth noting that biochar is typically produced through the thermochemical conversion of various biomass resources, such as agricultural residues, forestry waste, or sewage sludge, under oxygen-limited conditions, resulting in a carbon-rich material with a developed porous structure and abundant surface functionalities. Their study highlighted that the incorporation of metal oxides onto biochar not only enhances catalytic activity but also significantly improves catalyst stability and reusability over multiple cycles. Furthermore, these composites showed excellent reduction efficiency of 4-NP in aqueous media, confirming their potential as sustainable and cost-effective solutions for wastewater treatment. These findings strongly support the development of biochar-based catalytic systems as promising materials for environmental remediation applications [31]. Similarly, the study of Sonia Żółtowska et al. on three-dimensional commercial sponge-derived Co_3_O_4_@C catalysts demonstrated that carbon-based supports with highly porous architectures can effectively disperse Co_3_O_4_ nanoparticles, leading to enhanced accessibility of active sites and improved catalytic performance toward the degradation of organic contaminants. This further confirms the crucial role of carbonaceous matrices in stabilizing metal oxides and boosting their catalytic efficiency [32].

The Co_3_O_4_/biochar composite achieves its highest performance level through the dual process of optimizing its catalytic system and reaction conditions. The present study, therefore, employs a systematic experimental strategy based on statistical design tools to evaluate and optimize the influencing parameters governing the reduction of 4-nitrophenol. The response surface methodology (RSM) experimental design methods use statistical tools to assess how major elements impact the response of a particular system. The approach needs fewer experiments to produce accurate results because it maintains high reliability and accuracy and reproducibility of outcomes. The central composite design (CCD), together with the Box–Behnken design (BBD), serves as the main experimental design method that researchers use to optimize operational settings while they study how different factors interact with each other.

Several studies have successfully applied response surface methodology (RSM) for the optimization of reduction processes, particularly in the case of 4-nitrophenol (4-NP). For instance, the study entitled “Experimental Design for Optimization of 4-Nitrophenol Reduction by Green Synthesized CeO_2_/g-C_3_N_4_/Ag Catalyst Using Response Surface Methodology” employed a central composite design (CCD) to systematically investigate the effects of key operational parameters, including initial 4-NP concentration, catalyst dosage, reaction time, and NaBH_4_ amount [33].

Furthermore, another relevant study entitled “Experimental Design for Optimization of 4-Nitrophenol Reduction by Green Synthesized CeO_2_/g-C_3_N_4_/Ag Catalyst Using Response Surface Methodology” by Dephan Pinheiro et al. also confirmed the efficiency of RSM, where a central composite design (CCD) was applied to optimize the reduction process [33].

The present study demonstrates complete support for a circular economy approach that transforms urban sludge from wastewater into valuable industrial materials. The research introduces an original method that uses an uncommon biomass material to produce biochar that improves the material’s porosity and Co_3_O_4_ particle distribution. The initial material underwent acid pretreatment with HCl to extract potentially hazardous metals, which improved its environmental application capabilities. Researchers conducted a detailed study to understand how Co_3_O_4_ doping affects the structural and chemical characteristics of the material. The results demonstrate that Co_3_O_4_ incorporation causes major changes in material structure while it improves the process of carbon reduction to gas during thermal treatment. The process increases reactivity while it speeds up the progress of chemical reactions.

The experimental design approach enabled systematic optimization of operating conditions, which tested the catalytic reduction process that converted 4-nitrophenol (4-NP) into 4-aminophenol (4-AP). The research presents a unique contribution to the literature through its implementation of response surface methodology, which uses biochar derived from urban sludge as a new catalytic support method for 4-NP reduction. The study initiates a scientific exploration that develops sustainable environmental solutions through waste valorization and biochar property engineering and targeted catalytic application. To characterize the Co_3_O_4_/biochar nanocomposite, researchers used various techniques, which included X-ray diffraction (XRD), Fourier transform infrared spectroscopy (FTIR), scanning and transmission electron microscopy (SEM and TEM), UV-visible spectroscopy, pH_pzc_ determination, CHNS elemental analysis, and BET surface area analysis.

## 2. Results and Discussion

### 2.1. Materials Characterization

**Figure 1:**
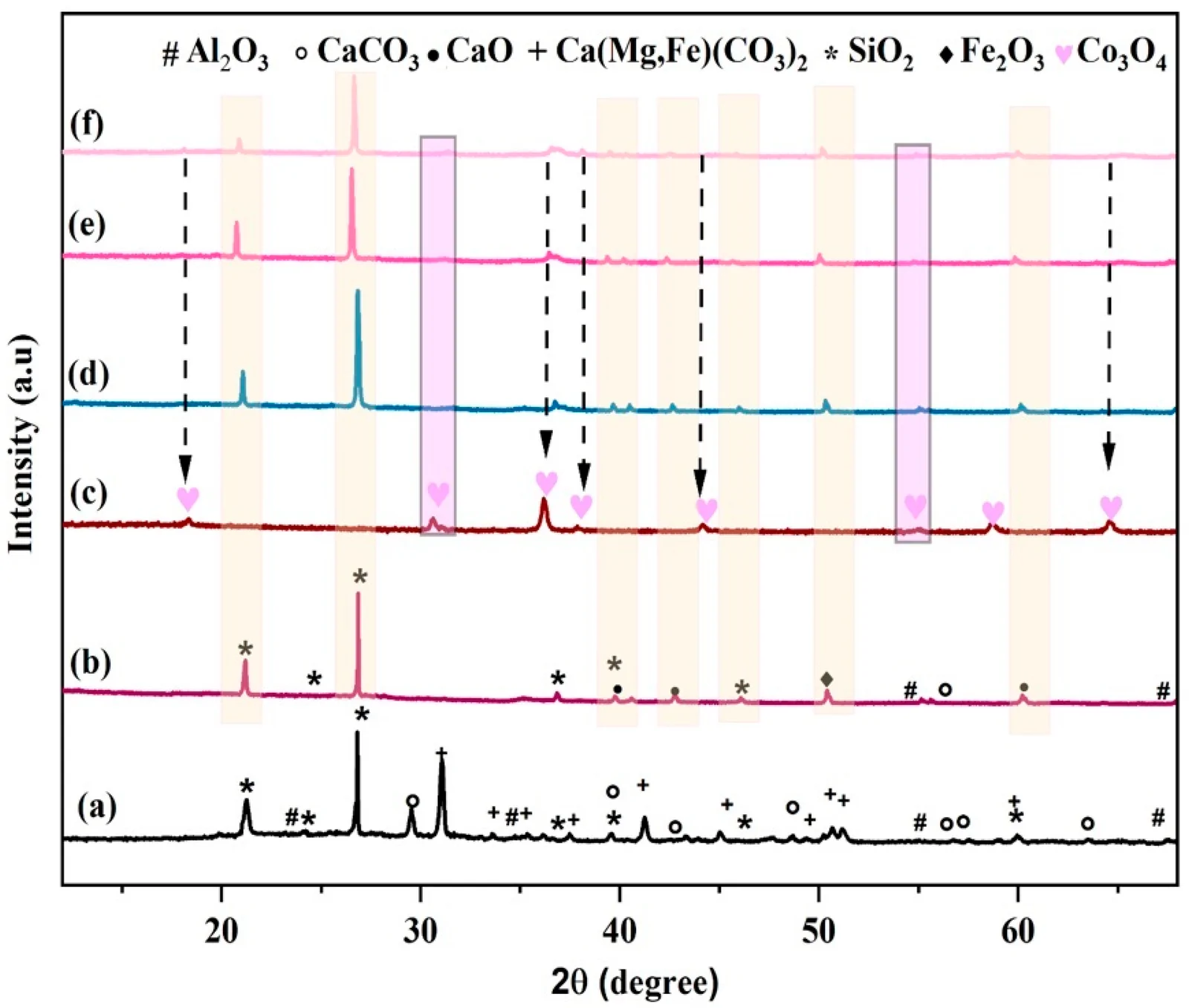
XRD patterns of (a) sewage sludge (SS), (b) biochar (BC), (c) cobalt oxide (Co_3_O_4_), and (d,e,f) xCo_3_O_4_/biochar composites with varying Co loadings (5%, 12.5%, 20%).

Figure 1 shows the XRD patterns of sewage sludge (SS), biochar (BC), cobalt oxide (Co_3_O_4_), and xCo_3_O_4_/biochar composites at various loading percentages (5%, 12.5%, and 20%). The analysis of SS confirmed the existence of mineral and organic matter. The quartz (SiO_2_), dolomite (Ca(Mg,Fe)(CO_3_)_2_), and calcium carbonate (CaCO_3_) were identified by the peaks at 20.84°, 26.56°, 30.88°, and 2θ = 29.36°, respectively [34]. The derivation of CaCO_3_ is probably from the lime used in sludge dewatering, while SiO_2_ comes from clay and sand residues in the wastewater. Less prominent peaks at 23.04°, 34.68°, and 54.92° pointed to the presence of alumina (Al_2_O_3_) [34]. After pyrolysis treatment, Figure 1 indicates that a variety of minerals experienced considerable changes. Ca(Mg,Fe)(CO_3_)_2_ loses CO_2_ upon heating, converted to CaO and Fe_2_O_3_, and this radically changes the mineral composition of the carbonized product. Afterward, the HCl treatment was found to be effective in eliminating minerals from the pyrolyzed sludge, dissolving and taking away most of the rest of the minerals—such as carbonates, metal oxides, and alumina (Al_2_O_3_)—while quartz (SiO_2_) stayed intact [35]. This process reduces the ash content and inorganic impurities, enhancing the quality of the produced biochar.

In the XRD patterns of Co_3_O_4_ nanostructures, the observed peaks at 2θ = 19.01°, 31.32°, 36.8°, 38.61°, 55.75°, 59.45°, and 65.34° correspond to the (111), (220), (311), (222), (422), (511), and (440) planes of the cubic Co_3_O_4_ structure (JCPDS #42-1467) [36]. The absence of impurity peaks confirmed the high crystallinity and successful synthesis of cobalt oxide.

For the Co_3_O_4_/biochar composites (Figure 1d,e,f), diffraction peaks corresponding to both Co_3_O_4_ and BC were observed, which verifies the successful synthesis of the composites. Moreover, the intensity of the dominant biochar peak at 2θ = 26.56° decreases with increasing Co_3_O_4_ content, which indicates that Co_3_O_4_ was effectively coated on the biochar surface.

**Figure 2:**
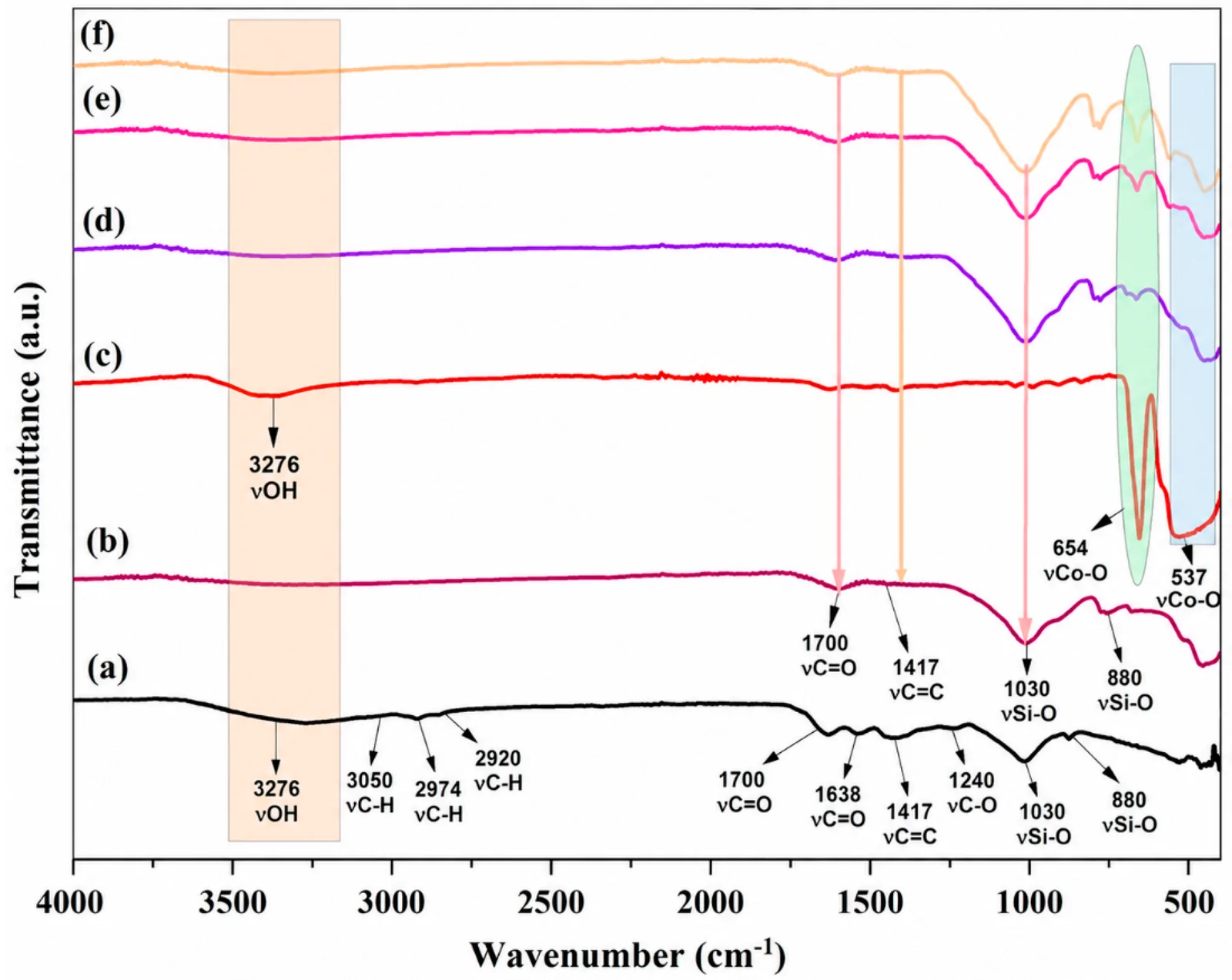
FTIR Analysis of (a) Sewage sludge (SS), (b) biochar (BC), (c) Co_3_O_4_, and (d,e,f) xCo_3_O_4_/biochar composites.

Figure 2 presents the FTIR spectra of SS, BC, Co_3_O_4_, and the composites with 5%, 12.5%, and 20% Co_3_O_4_ loading. The spectrum of SS (Figure 2a) reveals the presence of both organic functional groups and mineral phases, which align with the XRD results. The broad absorption band at 3276 cm^−1^ corresponds to O–H stretching vibrations from water, alcohols, phenols, or amines. Peaks observed at 3050, 2919, and 2844 cm^−1^ are attributed to C–H stretching vibrations of the glycidic portion of lipid content in the sludge. The band at 1700 cm^−1^ indicates C=O groups from lipids, while the peak at 1637 cm^−1^ is associated with protein structures (amide I). Additionally, the 1417 cm^−1^ peak corresponds to aromatic C–C stretching vibrations. A smaller band at 1240 cm^−1^ reflects C–O stretching vibrations from carboxylic acids. Mineral contributions are also evident, with bands at 450, 880, and 1030 cm^−1^ attributed to Si–O and Si–O–Si vibrations [37,38,39]. After pyrolysis and acid washing (Figure 2b), most of the organic-related peaks disappeared, while the SiO_2_-related peaks remained largely unchanged. Absorption bands at 1700 and 1417 cm^−1^ decreased significantly, reflecting the removal or degradation of some organic constituents.

The FTIR spectrum of the cobalt oxide sample (Figure 2c) exhibits two strong bands associated with Co–O stretching vibrations, which confirms the formation of spinel Co_3_O_4_ [40,41]. The first band (ν_1_) at 528 cm^−1^ corresponds to OB_3_ vibrations, where B represents Co^3+^ in octahedral sites, and the second band (ν_2_) at 652 cm^−1^ is related to ABO_3_ vibrations, with A corresponding to Co^2+^ in tetrahedral hole [42,43]. A large peak at 3363 cm^−1^ and a narrow one at 1639 cm^−1^ correspond to O–H stretching vibrations from adsorbed H_2_O molecules [44]. For the Co_3_O_4_/biochar composites (5%, 12.5%, and 20% Co_3_O_4_, Figure 2d,e,f), all characteristic peaks of biochar and Co_3_O_4_ are observed. Notably, the intensities of the Co^3+^–O and Co^2+^–O stretching vibrations increase with higher Co_3_O_4_ loading, which reflects the progressive incorporation of Co_3_O_4_ onto the biochar support.

**Figure 3:**
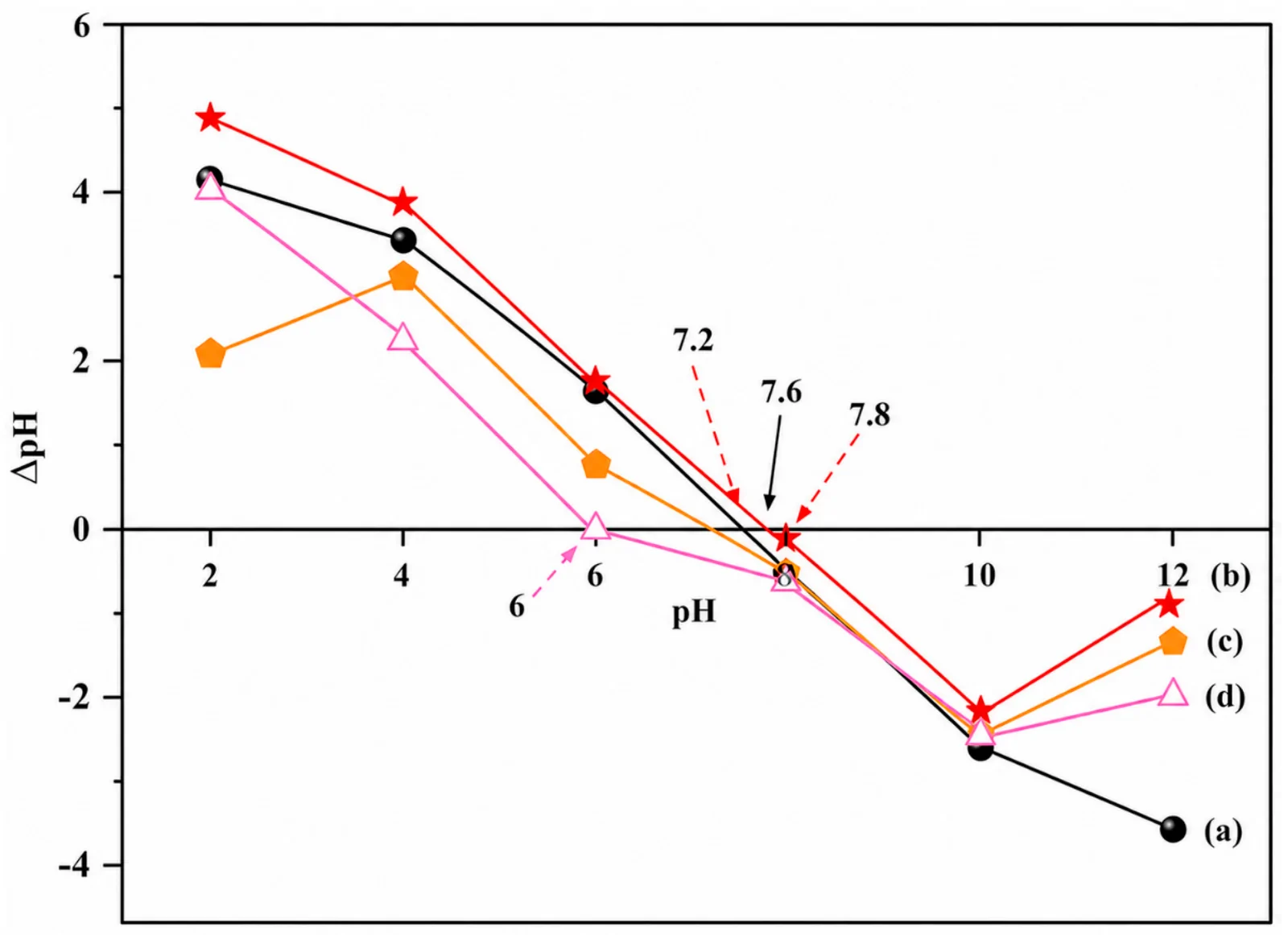
Determination of the point of zero charge of (a) sewage sludge, (b) biochar, (c) Co_3_O_4_, and (d) Co_3_O_4_/biochar composite.

The point of zero charge (pH_pzc_) of the sewage sludge, biochar, Co_3_O_4_, and Co_3_O_4_/biochar composite is illustrated in Figure 3. The sewage sludge displayed a pH_pzc_ of 7.2, which indicates that its surface charge is neutral at this pH value. The biochar presented a higher pH_pzc_ value of 7.8, which implies that the surface was mainly composed of basic functional groups. This might have been the result of the carbonization of organic matter and production of oxygen functions like hydroxyl (–OH) and carboxylate (–COO^−^). On the other hand, Co_3_O_4_ only, as per its intrinsic surface chemistry of the oxide, indicated a pH_pzc_ value of 7.5, which is just marginally lower compared to the biochar. Interestingly, the Co_3_O_4_/biochar composite revealed a pH_pzc_ value of around 6, confirming strong interactions between the biochar functional groups and Co_3_O_4_ [45]. Such interactions may be responsible for covering some oxygenated surface sites, resulting in the reduction of the composite material’s basicity. The above-mentioned change in surface charge properties is crucial, as it plays an important role in electrostatic interactions with pollutant ions in solution, thereby improving the material’s catalytic activity.

**Figure 4:**
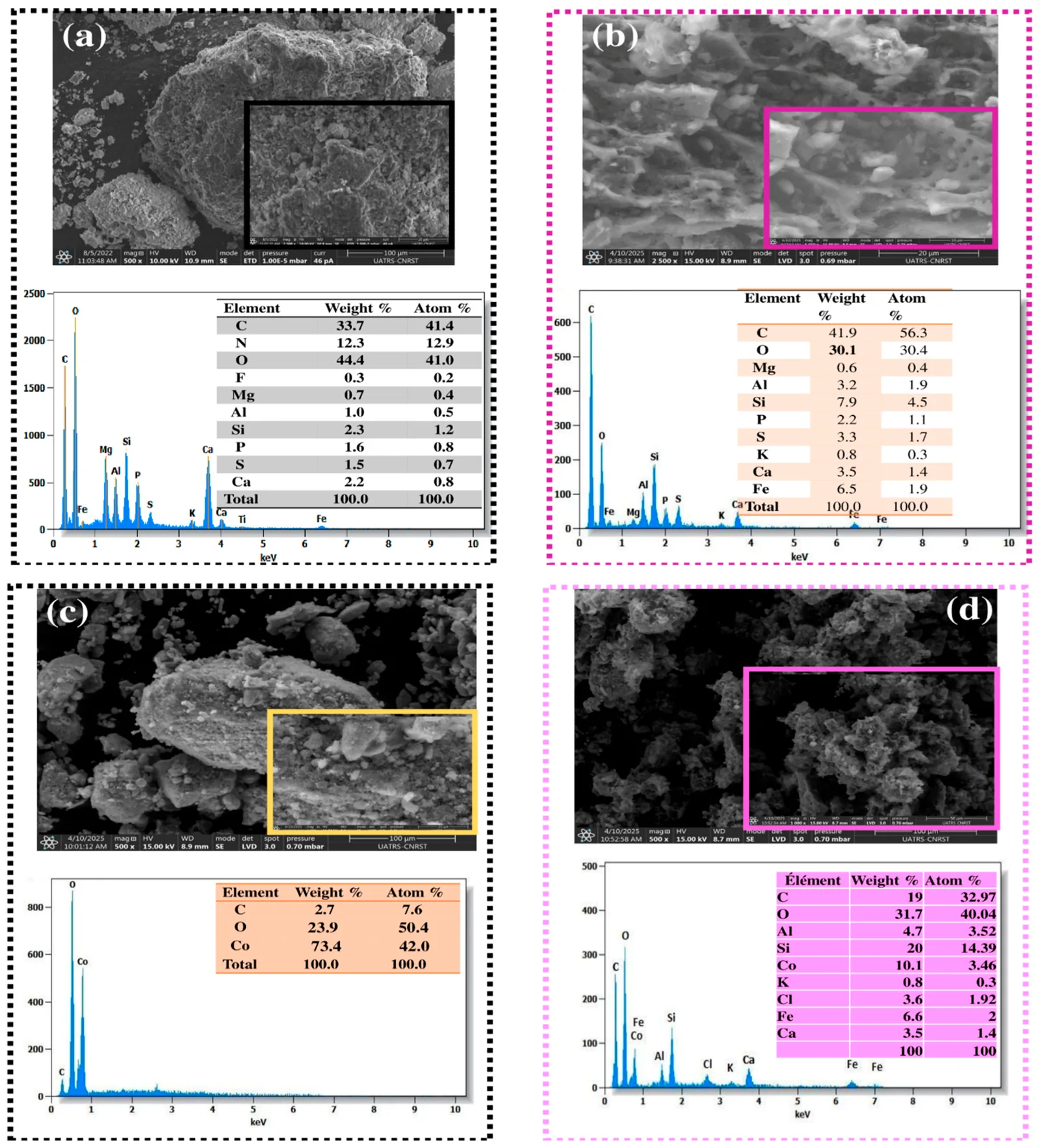
SEM images and EDX elemental maps of (**a**) sewage sludge, (**b**) biochar, (**c**) Co_3_O_4_, and (**d**) Co_3_O_4_/biochar composite.

To identify the chemical composition and morphology of the sewage sludge, biochar, and the Co_3_O_4_/biochar composite, the SEM/EDX was employed. As depicted in Figure 4a, the SEM image of sewage sludge (SS) revealed a compact and rough surface structure that contains some impurities and irregularly aggregated, block-like particles [46]. The biochar structure (Figure 4b) exhibits a well-developed porous structure and significant surface porosity, characterized by a developing porous structure and considerable surface porosity, resulting from the thermal decomposition of organic matter and the demineralization of ash through acid treatment [47,48]. On the surface of the BC, tiny nucleated sites were observed, corresponding to acid-insoluble mineral phases such as silica, which are resistant to elimination through HCl washing.

EDX analysis of the sewage sludge revealed the presence of C, N, O, Si, and Ca as the principal elements. The EDX analysis of biochar conducted after thermal and acid treatment confirmed the rise in carbon content alongside a reduction in metal content. Co_3_O_4_ nanoparticles’ SEM image in Figure 4c consists of particles in the range of nanometers with an aggregated morphology, yet fine details cannot be observed using this technique. EDX analysis validated the presence of only Co metal and O, which matches the chemical formulation of Co_3_O_4_. The small amount of carbon (~2.7%) detected comes from the carbon grid used for SEM/EDX measurements.

The SEM image of the 12.5% Co_3_O_4_/biochar composite (Figure 4d) shows particulate features closely associated with the biochar matrix, with strong adhesion between the oxide and the support. EDX analysis reveals the presence of Co_3_O_4_ alongside the other elements of the biochar, confirming the successful preparation of the composite.

**Figure 5:**
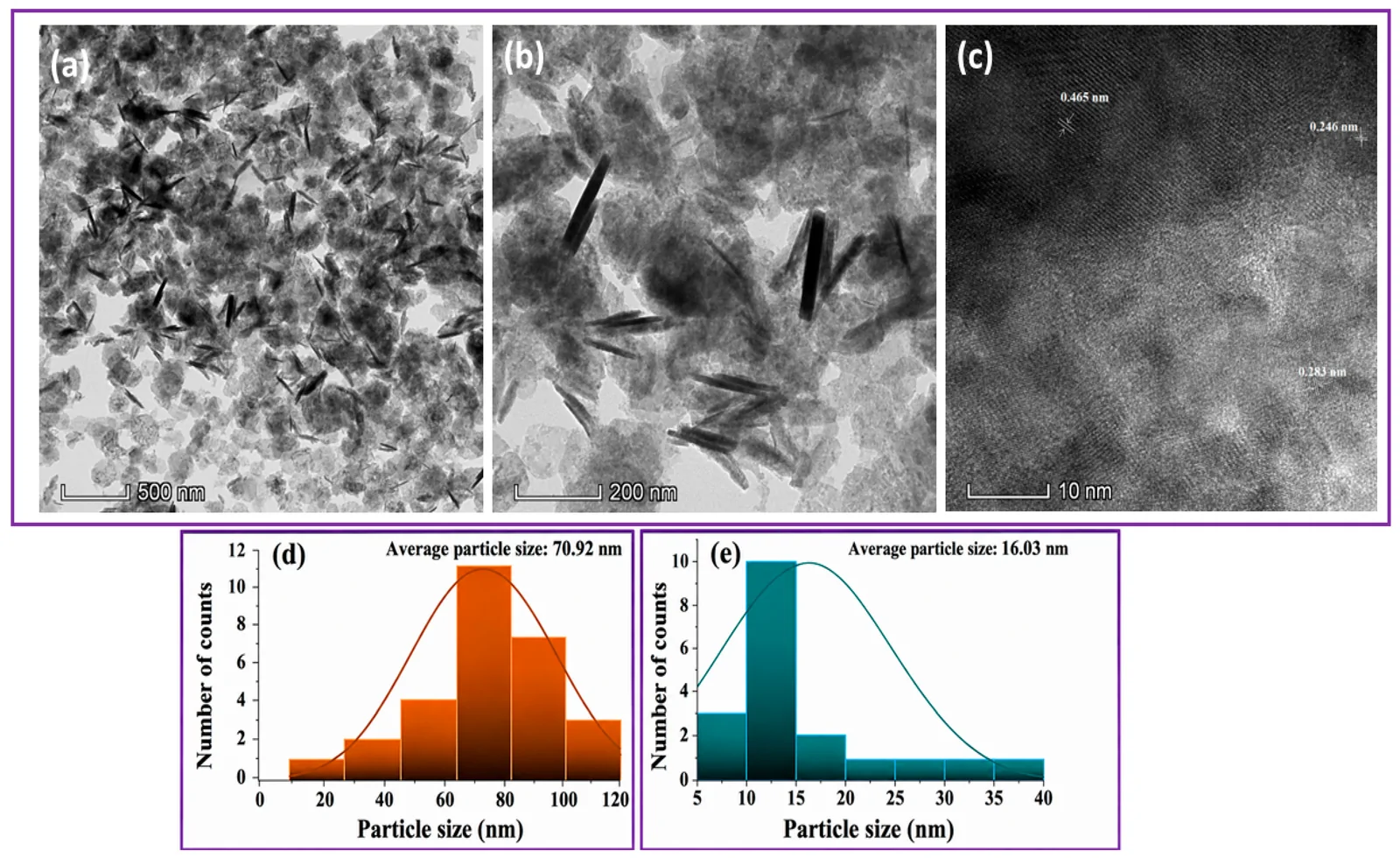
TEM images of Co_3_O_4_/biochar nanocomposite: (**a**,**b**) micrograph, (**c**) HR-TEM image, and (**d**,**e**) size distribution of hexagonal nanoplates (average edge length and thickness).

Figure 5 shows that the Co_3_O_4_ nanoparticles are evenly distributed throughout the surface of the biochar, forming distinct nanosheets [49,50]. This well distribution may facilitate and accelerate the catalytic reduction of 4-NP by improving the accessibility of active sites. Different orientations were displayed by the nanosheets. The majority of the nanosheets were in a face-on orientation, hence revealing a hexagonal shape, while others appeared edge-on, leading to a round-like morphology. The nanosheets, on average, had a diameter and a thickness of about 70.9 nm and 16 nm, respectively (Figure 5d and Figure 5e). The HRTEM image in Figure 5c depicts a clear view of the lattice fringes with interlayer spacings of 0.46, 0.28, and 0.24 nm, which are associated with the (111), (220), and (311) planes of cubic Co_3_O_4_, a finding corroborated by the XRD results [51].

**Figure 6:**
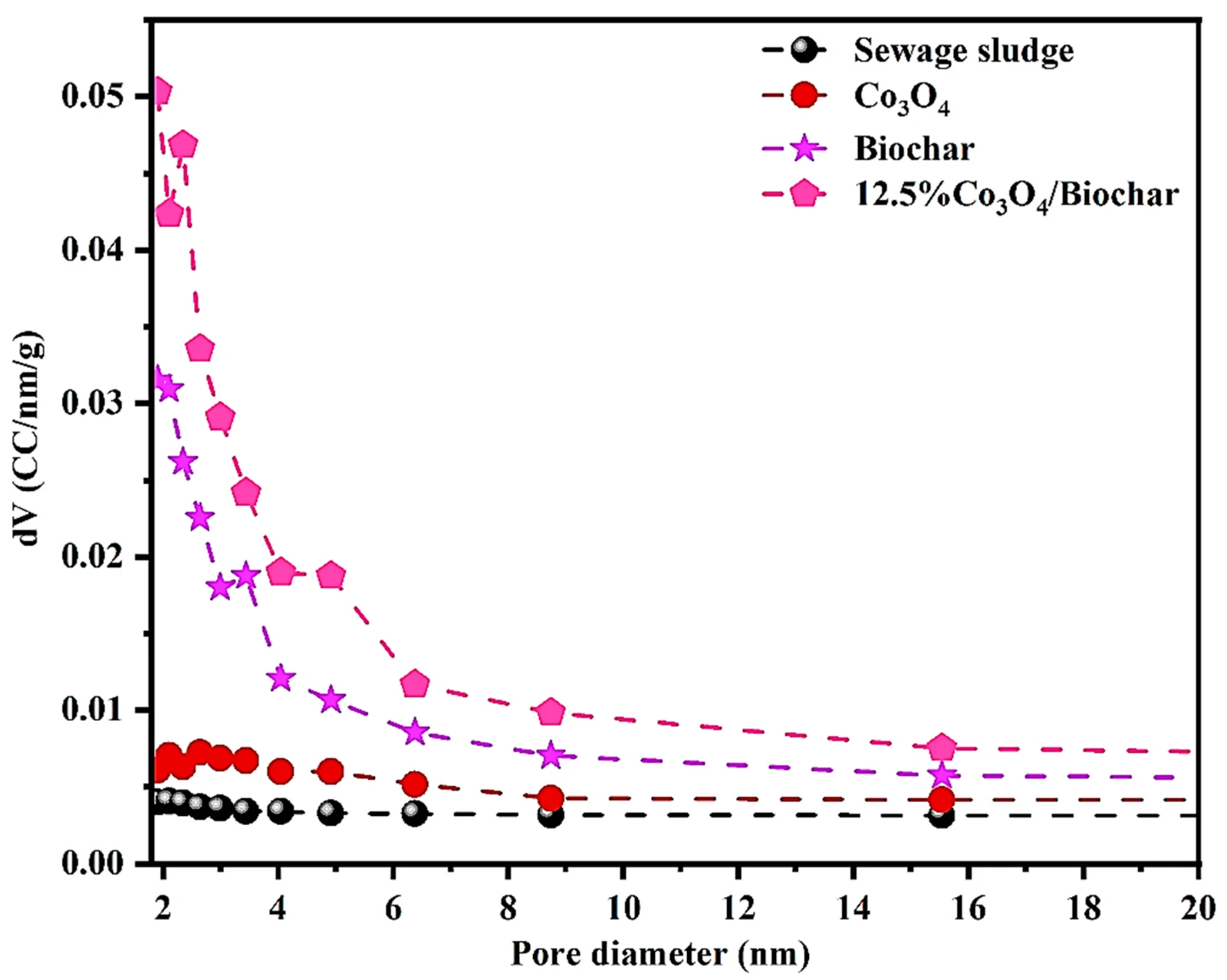
BJH pore size distribution of sewage sludge, Co_3_O_4_, biochar, and 12.5% Co_3_O_4_/biochar composite.

N_2_ adsorption–desorption analysis was conducted to evaluate the textural properties of the prepared materials. The specific surface area (SSA) was determined using the BET method, while the BJH method was employed to evaluate the pore size distribution. As summarized in Table 1, the textural properties were substantially improved following pyrolysis and subsequent modification. The SSA increased from 3.5 m^2^/g for sewage sewage sludge to 50.4 m^2^/g for biochar, which can be attributed to the thermal decomposition of organic constituents and the development of a more porous carbonaceous structure. Interestingly, the 12.5% Co_3_O_4_/biochar composite exhibited an even higher SSA of 75.7 m^2^/g, compared with 50.4 m^2^/g for biochar and 22.2 m^2^/g for pure Co_3_O_4_. This enhancement is supported by the BJH pore size distribution shown in Figure 6, where the composite exhibits the highest pore-volume contribution, particularly in the small mesopore region around 2–4 nm, followed by a broader contribution extending to approximately 15–20 nm. In comparison, biochar displays a lower but still significant mesoporous contribution, whereas Co_3_O_4_ and sewage sewage sludge exhibit much weaker pore distributions.

The pore volume follows the same trend, increasing from 0.004 cm^3^/g for sewage sewage sludge and 0.084 cm^3^/g for biochar to 0.198 cm^3^/g for the 12.5% Co_3_O_4_/biochar composite. This substantial increase indicates the development of a more accessible porous network in the composite. The higher pore volume and the enhanced BJH pore size distribution suggests that the incorporation of Co_3_O_4_ into the biochar matrix modifies the textural structure and increases the contribution of mesopores. The simultaneous increase in surface area and pore volume is consistent with a more developed porous architecture, which can facilitate the diffusion of reactant molecules toward the catalytically active Co_3_O_4_ sites. Therefore, the combination of the carbonaceous matrix and Co_3_O_4_ results in a complementary textural structure, providing a larger accessible surface and improved molecular transport compared with the individual components [45,52].

**Figure 7:**
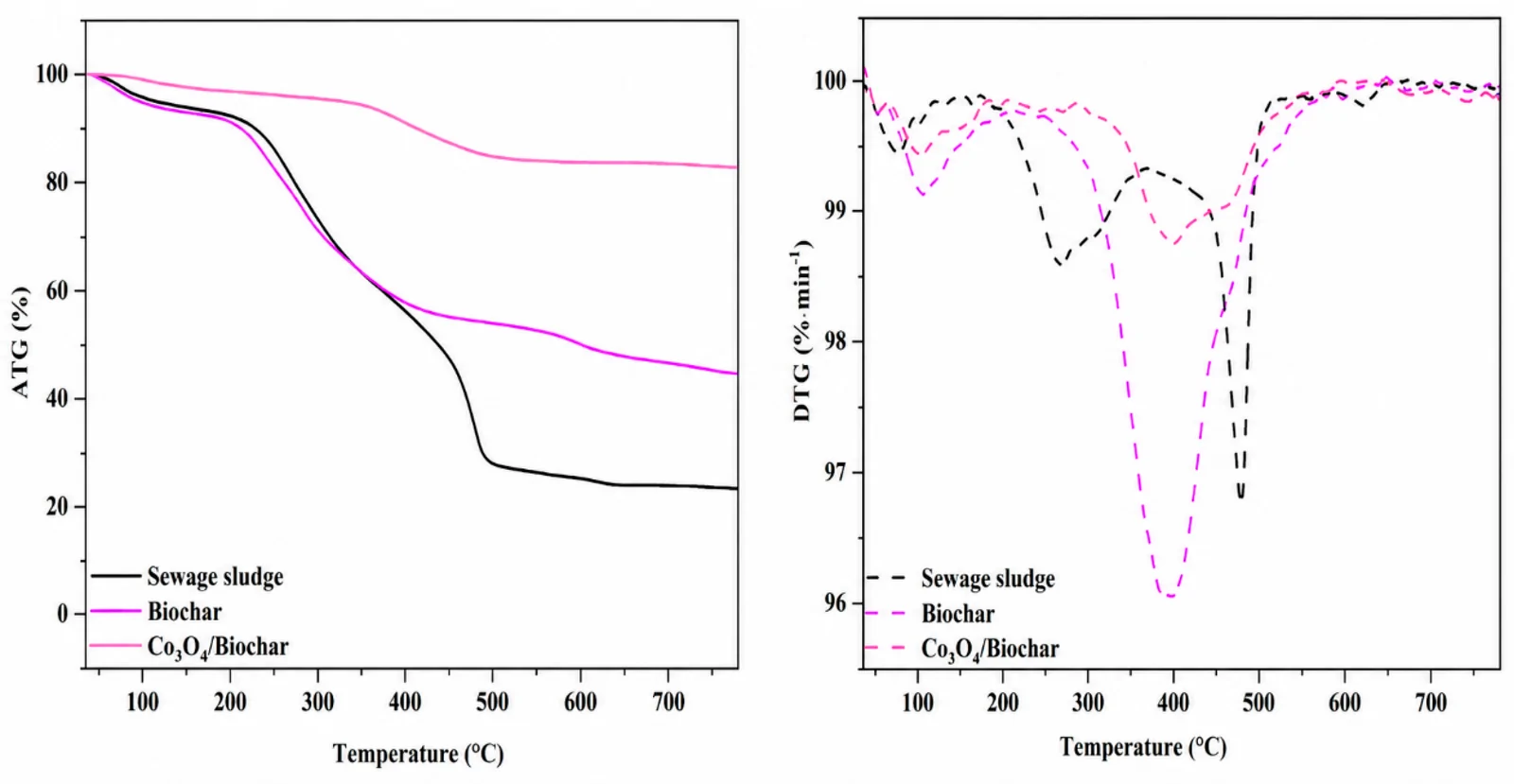
TGA/DTG curves of sewage sludge, biochar, and Co_3_O_4_/biochar composite under air.

The thermogravimetric analysis (TGA/DTG) of the sewage sludge, biochar, and Co_3_O_4_/biochar composite under an oxidative atmosphere, as illustrated in Figure 7, depicts different thermal profiles. The sewage sludge presents a total mass loss of 75.9%, with an initial loss at about 84.32 °C due to moisture removal. After that, the major thermal degradation observed in the range 200 °C to 517 °C may correspond to the decomposition of complex organic matter. The last peak seen between 517 °C and 646.54 °C corresponds to the thermal degradation of CaCO_3_ into CaO and CO_2_. The total mass loss for the biochar is 51.48%, as illustrated in Figure 6. The loss of mass at a temperature of 107.41 °C can be attributed to the process of dehydration. The degradation between 200 °C and 418 °C is related to the process of oxidation of carbon during thermogravimetric analysis in the presence of air. The last peak, which occurs between 517 °C and 646.54 °C, is due to the continued decomposition of remaining CaCO_3_. For the Co_3_O_4_/biochar composite, the maximum mass loss recorded is 43.36%. An initial dehydration step is also observed at 107.41 °C, followed by a significant degradation in the temperature range of 281–418 °C, related to the oxidation of carbon in air. After Co_3_O_4_ incorporation and the residual presence of CaCO_3_, the Co_3_O_4_/biochar composite exhibits improved thermal stability compared to the biochar alone [53,54].

The CHNS elemental analysis (Table 2) confirms the observed results from TGA and SEM. The pyrolysis process leads to a considerable increase in carbon content (from 33.82% for sewage sludge to 42.05%; this is a direct indication of the accumulation of carbonaceous structures and the elimination of volatile substances. Simultaneously, the sulfur content drops drastically (from 0.77% to 0.13%), indicating effective desulfurization during the thermal treatment. The subsequent introduction of Co_3_O_4_ leads to a reduction in carbon content to 29.8%, which may be attributed to the oxidation of the most labile carbon fractions during calcination in an oxidizing atmosphere and to the dilution effect caused by the incorporation of the inorganic cobalt oxide phase. In addition, the nitrogen and sulfur contents increase slightly in the composite. This can be explained by the relative increase in their concentration due to the decrease in carbon, as well as by the stabilization or immobilization of nitrogen- and sulfur-containing species in the presence of cobalt [55].

### 2.2. Catalytic Tests

#### 2.2.1. The Effect of Co_3_O_4_ Content on the Catalytic Reduction

**Figure 8:**
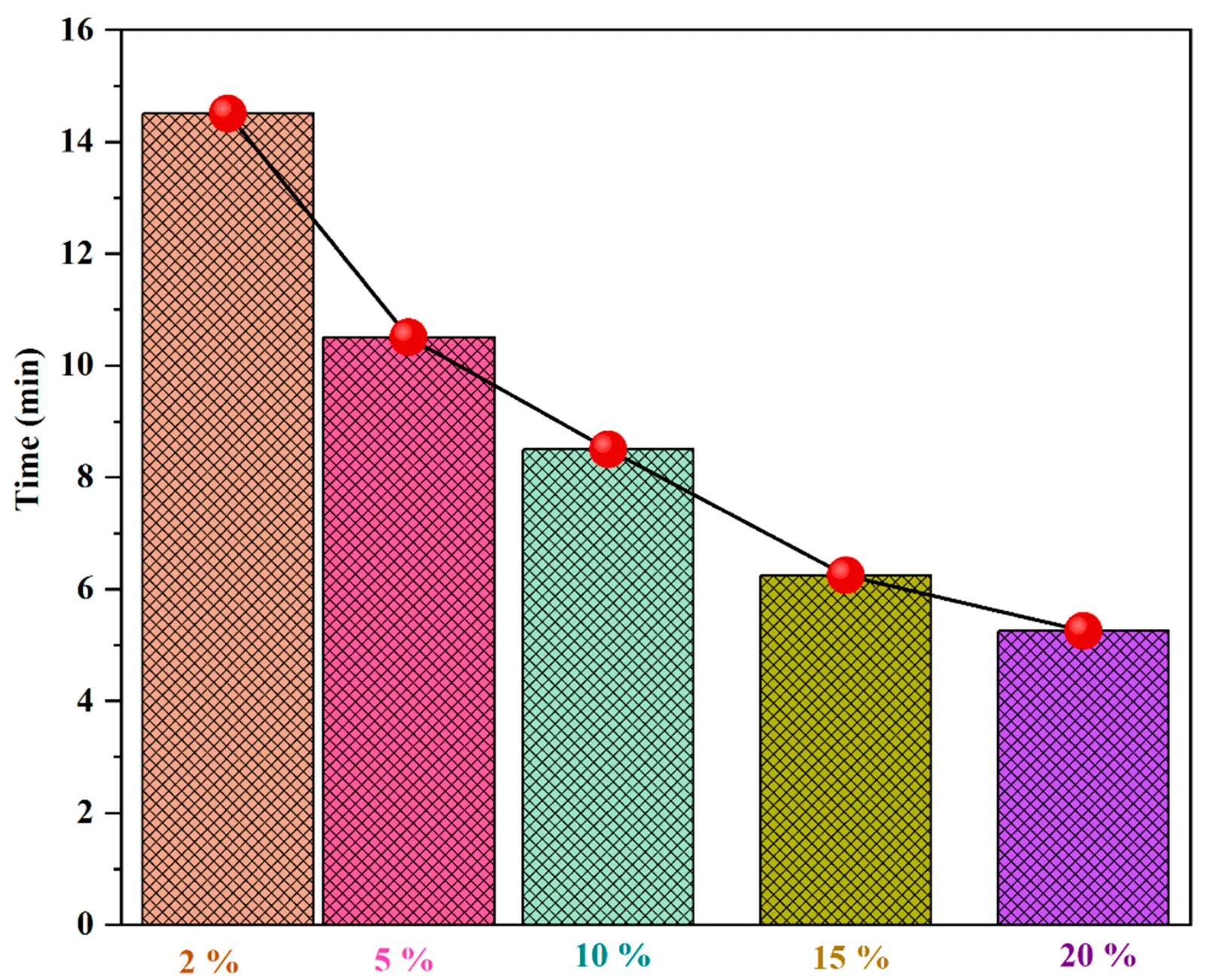
Influence of Co_3_O_4_ loading on the time of catalytic reduction of 4-NP, conditions of reaction: 6 mg of Co_3_O_4_/biochar catalysts, [NaBH_4_] = 18 mM.

To investigate the effect of the Co_3_O_4_ content in the composite on the catalytic reduction of 4-NP, a series of catalysts with varying Co_3_O_4_ loadings (2%, 5%, 10%, 15%, and 20%) were synthesized and tested. As illustrated in Figure 8, the time required to ensure complete reduction of 4-NP decreases progressively as the Co_3_O_4_ content increases. This behavior can be related to the higher density of active Co_3_O_4_ sites on the surface of catalysts with greater loadings. The presence of an increasing number of active sites results in a more efficient electron transfer. In addition, the higher oxide content markedly increases the surface area of the composite, accelerating the interaction between the catalyst and the reactant. These findings indicate a positive correlation between the amount of Co_3_O_4_ and the catalytic performance. Furthermore, they reveal the availability of very active sites as the most important factor facilitating the 4-NP reduction kinetics, hence providing a setup for the determination of the optimal Co_3_O_4_ content for rapid and efficient catalytic conversion [56].

#### 2.2.2. The Effect of NaBH_4_ Concentration on the Catalytic Reduction of 4-NP

**Figure 9:**
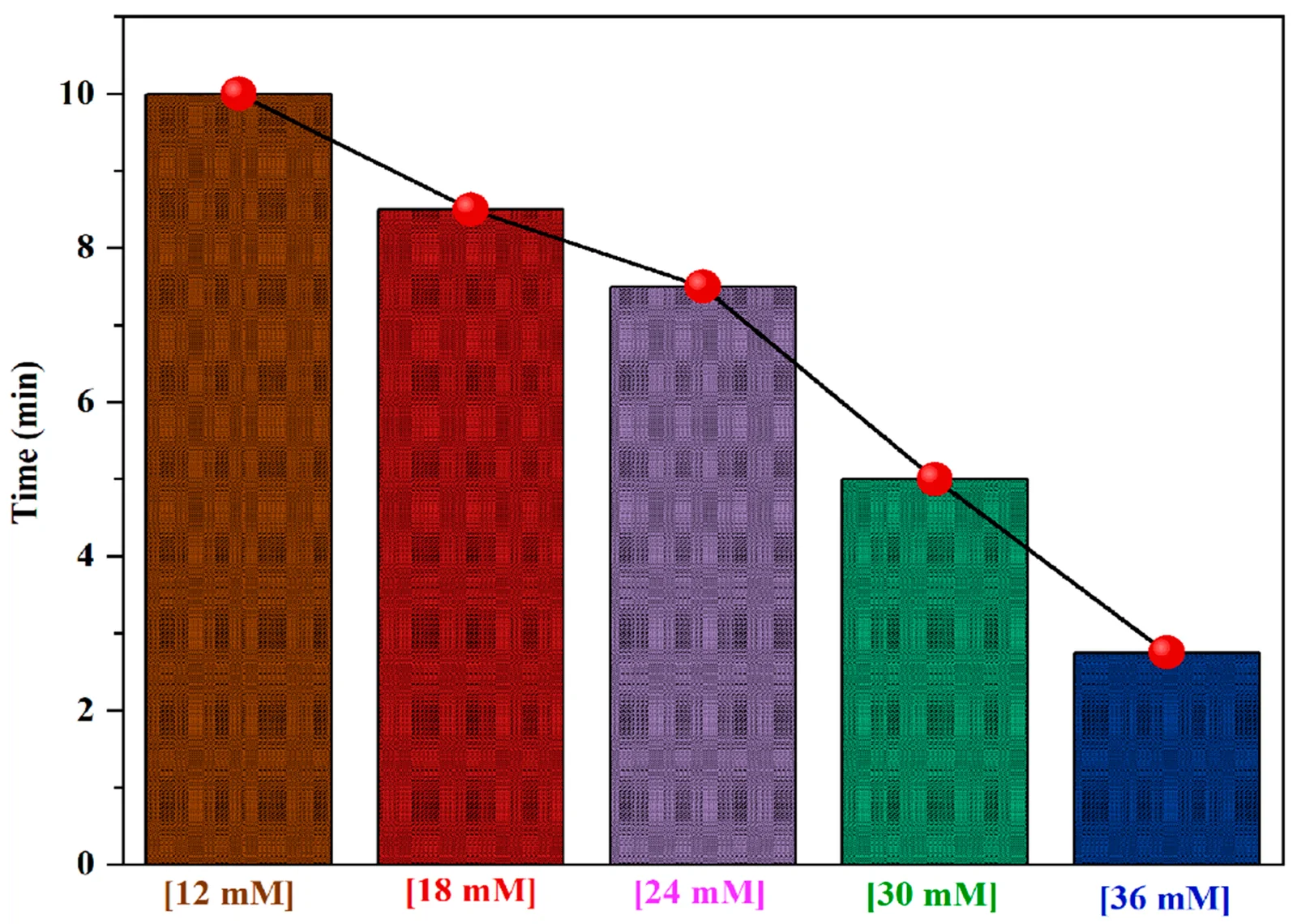
Effect of NaBH_4_ concentration on the time of catalytic reduction of 4-NP. Reaction conditions: 6 mg of 10%Co_3_O_4_/biochar catalyst.

The influence of NaBH_4_ concentration on the catalytic performance of cobalt oxide catalysts was investigated through a sequence of reduction experiments wherein the concentration of NaBH_4_ was varied between 12 mM and 36 mM while the catalyst loading and Co_3_O_4_ content were held constant. The time required for the complete reduction of 4-NP, indicated in Figure 9, decreases considerably as the amount of NaBH_4_ is increased. At 12 mM, the time for reduction was about 10 min, but at 36 mM, the reaction was finished in around 2.8 min. This can be explained by the fact that higher NaBH_4_ concentrations lead to more active hydrogen species being present, which in turn accelerates the electron transfer to 4-NP, thus increasing the reduction rate. The inverse dependence of reducing agent concentration and reaction time agrees with previous literature, confirming that NaBH_4_ concentration plays a crucial role in optimizing the catalytic reduction process [56].

#### 2.2.3. The Effect of Catalyst Dosage on the Catalytic Reduction

**Figure 10:**
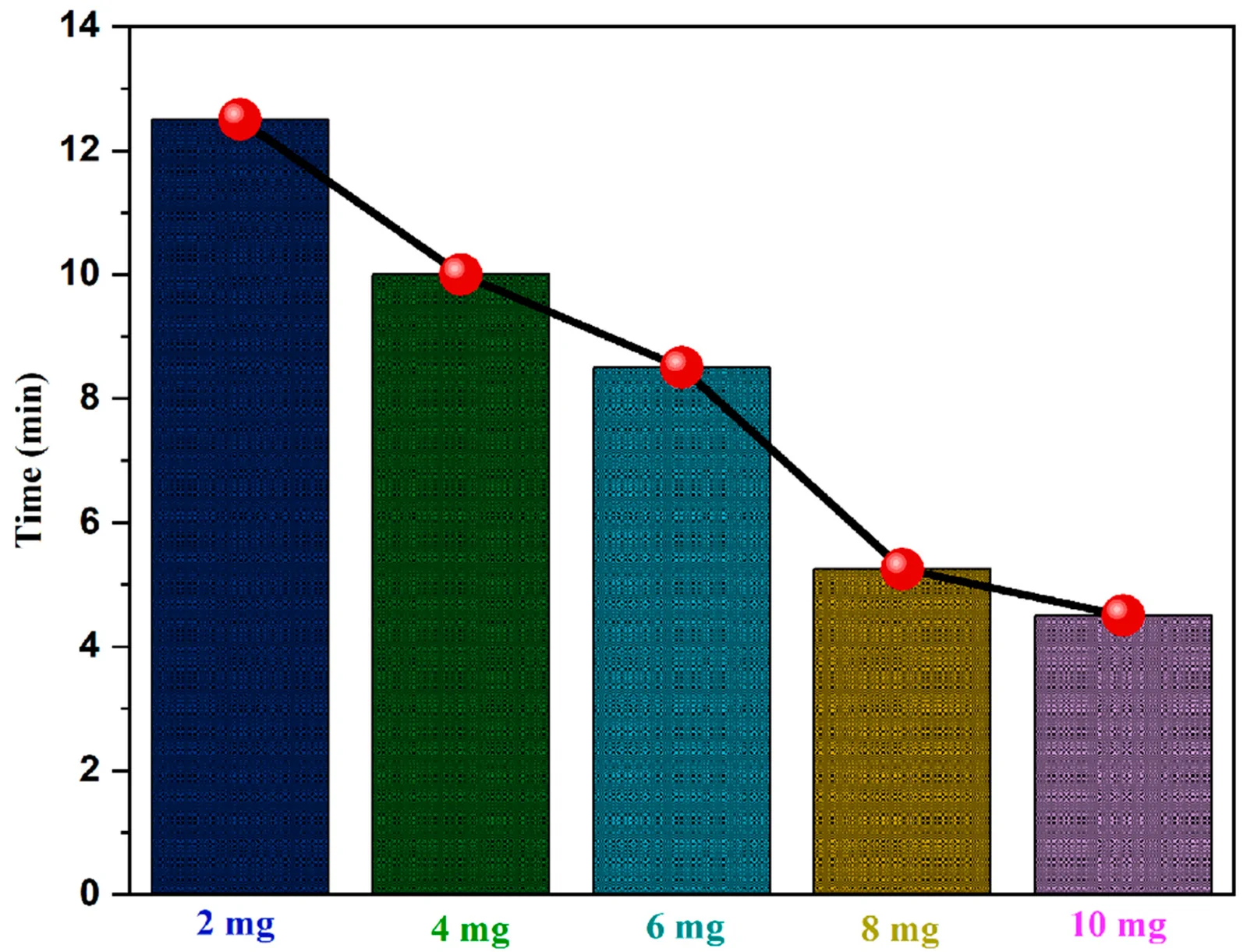
Effect of catalyst amount on the time of catalytic reduction of 4-NP, reaction conditions: 10%Co_3_O_4_/biochar catalyst, [NaBH_4_] = 18 mM.

It is well established that the reduction of 4-NP by NaBH_4_ cannot take place without a catalyst [57]. To examine how the catalyst amount affects the reaction, catalysts with identical Co_3_O_4_ loadings were tested at different weights. Figure 10 shows that lower catalyst mass significantly increases the reduction time, while higher catalyst dosage leads to faster reaction and less time needed for complete 4-NP reduction. This phenomenon can arise from the relationship between the amount of catalyst and the number of available active sites. The adsorption of 4-NP molecules and BH_4_^−^ ions onto the catalyst surface is limited when the catalyst amount is low because fewer active sites are exposed to the reactant. As a result, fewer effective collisions between reactants and catalytic sites occur, reducing the overall reaction rate. Conversely, along with increasing catalyst mass, the total surface area and the number of accessible active sites increase. This improved surface availability allows more reactant molecules to interact with the catalyst, thereby accelerating the electron transfer from BH_4_^−^ to 4-NP, resulting in faster conversion of pollutant. Moreover, a higher catalyst loading can improve the dispersion of reactants on the surface, reducing mass-transfer limitations and ensuring that the reduction proceeds more efficiently [58].

### 2.3. Optimization of 4-Nitrophenol Reduction via Response Surface Methodology (RSM)

#### 2.3.1. Statistical Model Analysis

The optimization of the 4-NP reduction to 4-AP was performed through the RSM using the central composite design (CCD). This undertaken approach evaluated three variables: the catalyst dosage (X_1_), the concentration of NaBH_4_ (X_2_), and the loading of Co_3_O_4_ in the composite (X_3_). Design experiment containing 19 tests led to a comprehensive assessment of the impacts of these factors on the reduction time (Y); the full experimental matrix and the observed responses are presented in Table 3.

The polynomial quadratic model representing the correlation between the estimated response (4-NP reduction time) and the process parameters is as follows:

(1) $$Y=5.48-3.13X_{1}-2.28X_{2}-3.3X_{3}+0.375X_{1}X_{2}+0.75X_{1}X_{3}+0.1875X_{2}X_{3}\;+1.71{X_{1}}^{2}+1.24{X_{2}}^{2}+2.11{X_{3}}^{2}$$

Equation (1) obtained states that the reduction time of 4-NP is inversely related to the catalyst dose (X_1_), NaBH_4_ concentration (X_2_), and Co_3_O_4_ mass percent (X_3_), thus speeding up the reaction kinetics. Consequently, these parameters positively influence the reduction process, despite the negativity of their linear coefficients. On the other hand, the positive coefficients of the interaction terms among the three parameters (X_1_X_2_, X_1_X_3_, and X_2_X_3_) imply that the simultaneous increase of two parameters extends the reaction time, which is opposite to their individual effects. The strongest interaction is noticed between the catalyst mass and the oxide content (+0.7700), implying that the use of a large amount of catalyst with a high percentage of oxide causes the particles to agglomerate, decreases the number of active sites, and results in limitations of mass transfer, hence the rate of catalytic reaction decreases.

Additionally, the quadratic terms associated with the dose of catalyst (X_1_^2^), NaBH_4_ concentration (X_2_^2^), and Co_3_O_4_ loading in the composite (X_3_^2^) also have positive coefficients, which means there is a convex curve and the existence of an optimal level for each parameter. The content of the oxide (X_3_^2^) shows the most marked curvature, indicating that precise control over the metal oxide content is vital for the reduction time optimization.

Analysis of variance (ANOVA) is a statistical technique used in experimental design to assess the quadratic polynomial model’s sufficiency and importance. The ANOVA results for the reduction of 4-NP are represented in Table 4. The model’s F-value of 46.13, along with a very low *p*-value (<0.0001), confirms that the model is of statistical significance and it has an excellent fit with the experimental data for the reduction of 4-NP over time. The analysis of the main effects indicated three factors that are statistically significant (*p* < 0.05): catalyst dosage (X_1_), NaBH4 concentration (X_2_), and weight percent of Co_3_O_4_ (X_3_). This indicated that all variables X_1_, X_2_, and X_3_ significantly affect the time for the conversion of 4-NP to 4-AP in the following order: Co_3_O_4_ loading **>** catalyst dosage **>  **NaBH_4_ concentration.

Notably, one key interaction effect was identified between the catalyst dosage and the Co_3_O_4_ content (X_1_X_3_). Additionally, two quadratic terms, catalyst dosage (X_1_^2^) and Co_3_O_4_ weight content (X_3_^2^), exhibited significant influence, which collectively validates the appropriateness of the quadratic polynomial model for describing the complex relationships between the process variables. The F-value of lack of fit (2.18 in Table 4) suggests that the lack of fit is not significant relative to the pure error, which suggests that the selected regression equation is adequate to describe the relationship between the reduction time of 4-NP and the parameters X_1_, X_2_, and X_3_. Therefore, the model was considered valid for the present work. The data in Table 5 confirm a strong correlation between predicted and experimental values and an excellent predictive capability of the model for the 4-NP reduction time, which is supported by high R^2^ (0.9788) and adjusted R^2^ (0.9576) values [59].

**Figure 11:**
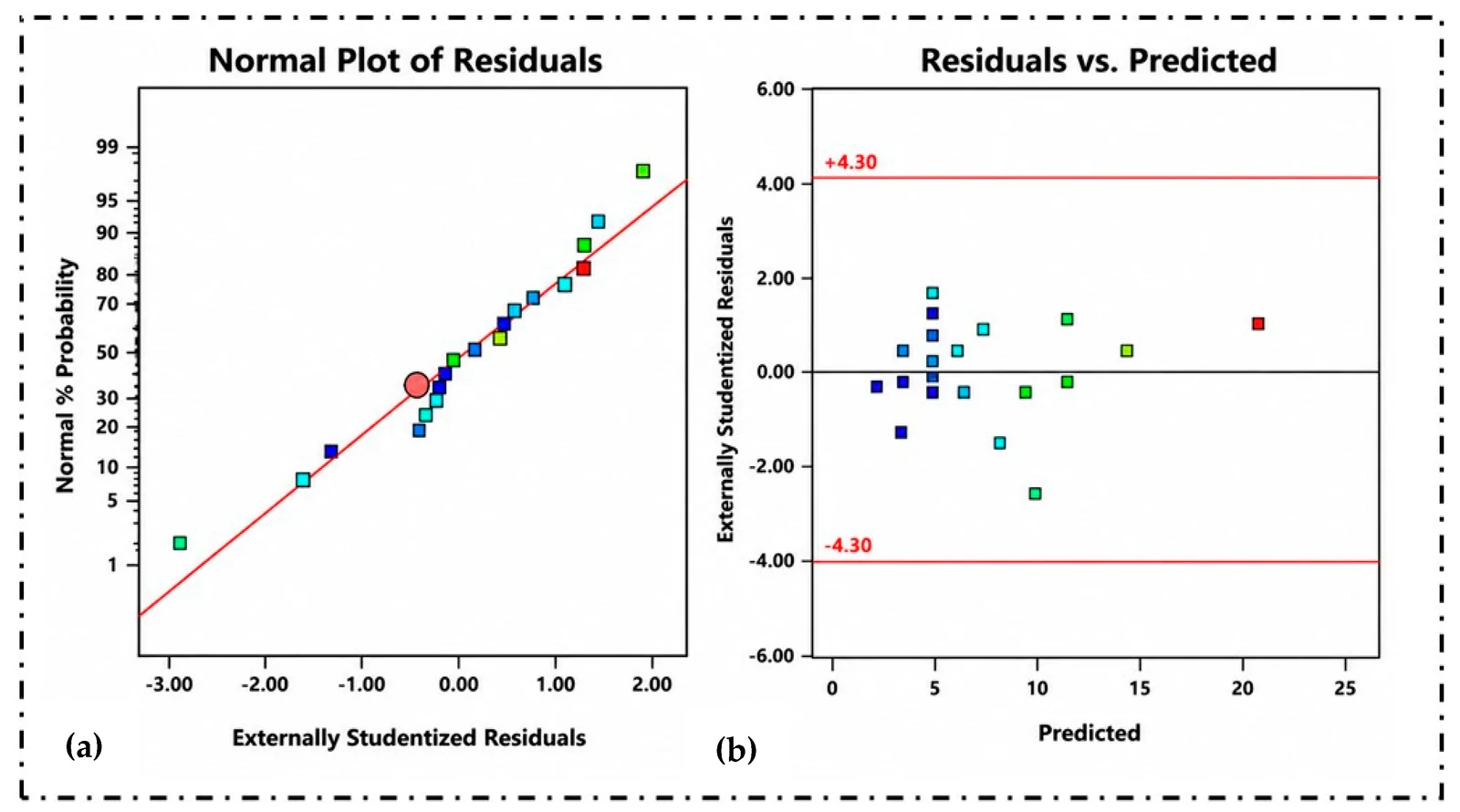
Validation of 4-NP reduction model: (**a**) predicted-observed correlation, (**b**) residual analysis by run sequence.

Figure 11a compares the actual response values with their corresponding model-predicted values. This predicted versus actual plot helps identify potential outliers or data clusters that may be challenging for the model to predict accurately. The distributed points closely align with the straight line, which reveals good matching between the observed and predicted values within the experimental range (Figure 11a). Furthermore, Figure 11b shows a random distribution of residuals across all experimental runs, which demonstrates no systematic deviation between the observed and predicted values. These results confirm the reliability of the quadratic polynomial model in describing the relationship between process variables and response [60].

#### 2.3.2. Response Surface and Contour Diagrams

**Figure 12:**
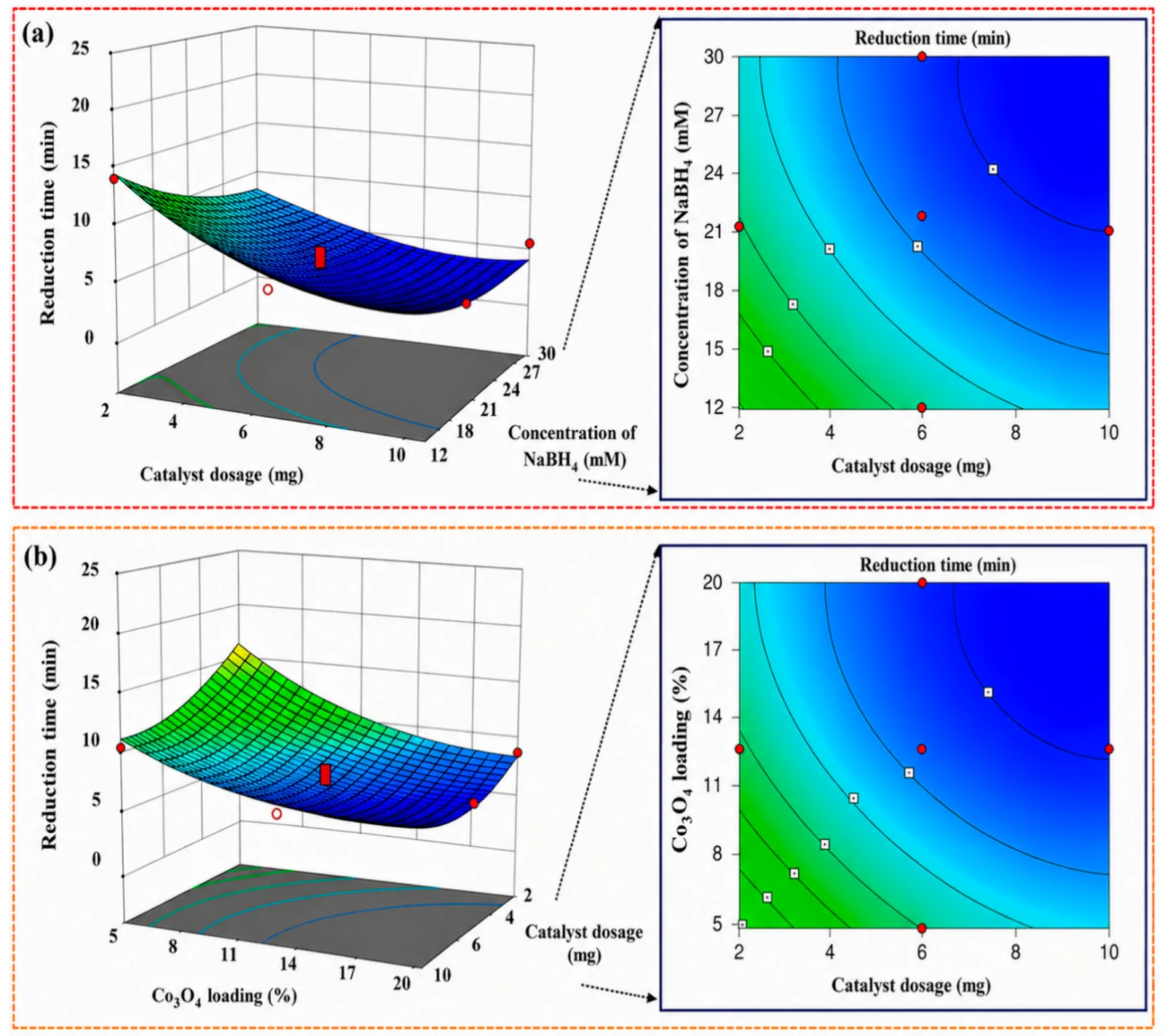
The 3D response surface figures and contour diagrams showing (**a**) the effect of catalyst dosage and NaBH_4_ concentration on the reduction time, and (**b**) the effect of catalyst dosage and Co_3_O_4_ loading on the reduction time.

The combined effect of two operating parameters on the reduction time (Y) of 4-NP to 4-AP is represented by the 3D response surface and 2D contour plots (Figure 12a,b), where the remaining variable is kept constant [58].

The combined effect of the catalyst dosage (X_1_) and NaBH_4_ concentration (X_2_) is illustrated in Figure 12a. When both the catalyst dosage and NaBH_4_ concentration are increased, a significant reduction in time is observed. This behavior can be attributed to the accessibility of more active sites as well as the excess of reducing power, both of which speed up the reaction.

Similarly, Figure 12b depicts the interaction between the catalyst dosage (X_1_) and the Co_3_O_4_ loading in the composite (X_3_). The results reveal that individually increasing either the catalyst dosage or the Co_3_O_4_ loading leads to a reduction in the reduction time; consequently, the reactivity of the system is improved. However, their interaction effect demonstrates a slightly different trend: the reduction time along the diagonal is less marked than their individual contribution. This is supported by the positive coefficient of the interaction term between X_1_ and X_3_ indicated by the ANOVA, which also indicates that this interaction is statistically significant (high *F*-value, *p* < 0.05) and stronger than the other interactions.

#### 2.3.3. Optimized Process Parameters for 4-NP Reduction

The developed model was able to describe the dependence of the reduction time of 4-NP on catalyst dosage, NaBH_4_ concentration, and Co_3_O_4_ content in the composite. Besides, it allowed the determination of the optimal operating conditions through the use of a multi-criteria numerical optimization technique that systematically assesses the design space to obtain balanced compromises among the involved parameters. We were using quality ramp diagrams, with the quality function (from 0, denoting out-of-limits conditions, to 1, signifying the ideal target) serving as the main performance indicator. Numerical optimization took into account all factors to find their optimal levels. The objective of this optimization was to select operating conditions that allow the reaction time to remain sufficient for detailed kinetic monitoring while avoiding excessively long durations [33,59].

**Figure 13:**
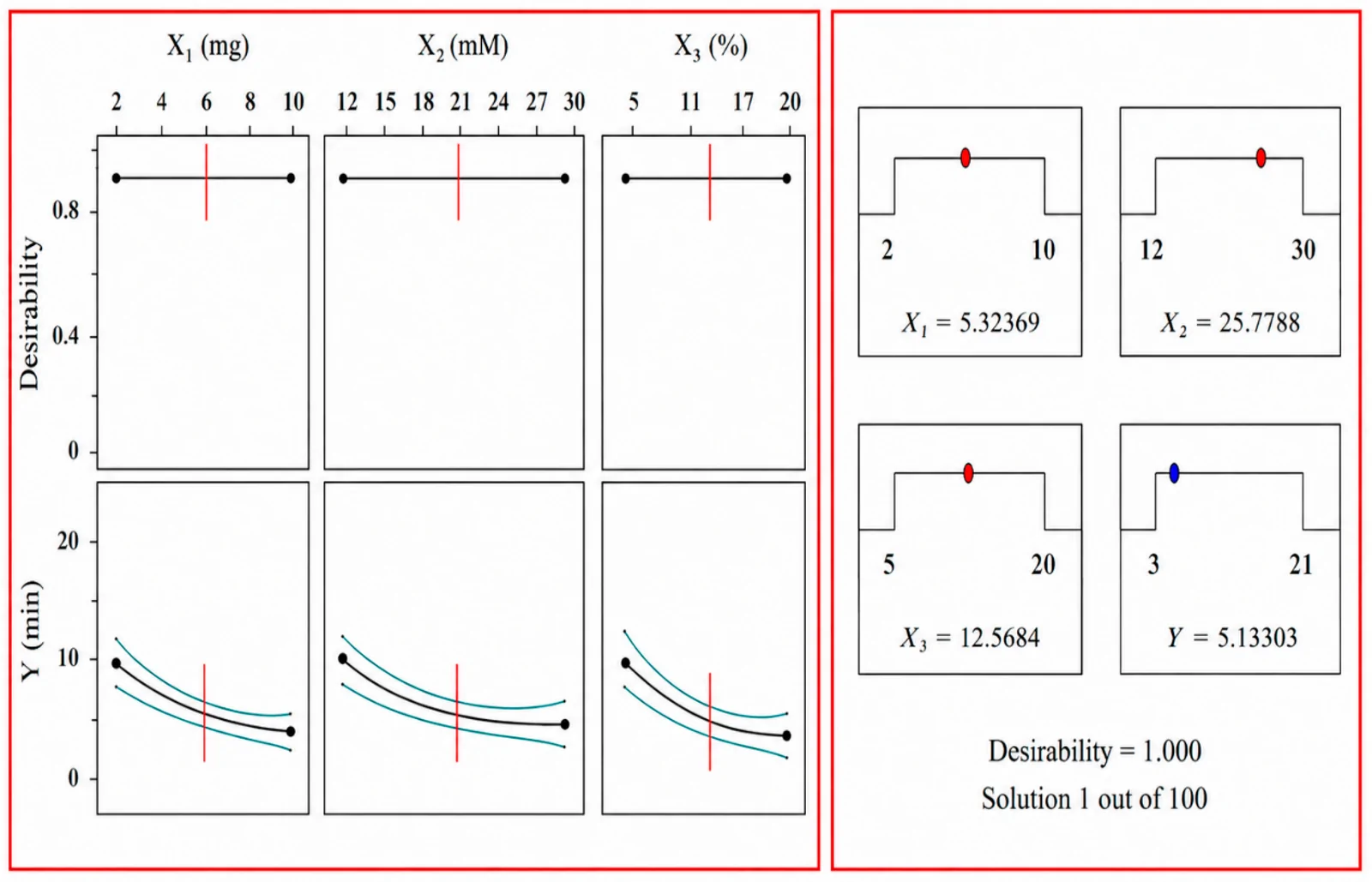
Numerical optimization results predicted by the RSM model.

Desirability ramp optimization results for the reduction of 4-NP to 4-AP in the presence of NaBH_4_ and Co_3_O_4_/biochar catalyst are shown in Figure 13. The best conditions were found to be a catalyst dosage (X_1_) of 5.32 mg, a NaBH_4_ concentration (X_2_) of 25.78 mM, and a Co_3_O_4_ content in the composite (X_3_) of 12.57%, which resulted in an optimum reduction time of about 5.13 min. The reported optimum corresponds to the best combination of the investigated operating parameters within the selected experimental domain, as predicted by the RSM model. A test was done to check the model’s predictive power under the mentioned optimal conditions. The reaction was finished in 5.5 min, which is very close to the predicted duration, thus confirming the reliability and accuracy of the developed model.

### 2.4. Kinetics Study of Catalytic Reduction of 4-NP Under the Optimal Conditions

Under the optimum conditions obtained from design experiment analysis, kinetic analysis of the heterogeneous reduction of 4-NP in the presence of NaBH_4_ and the Co_3_O_4_/biochar catalyst was performed additionally to highlight the catalytic performance of the synthesized composite.

**Figure 14:**
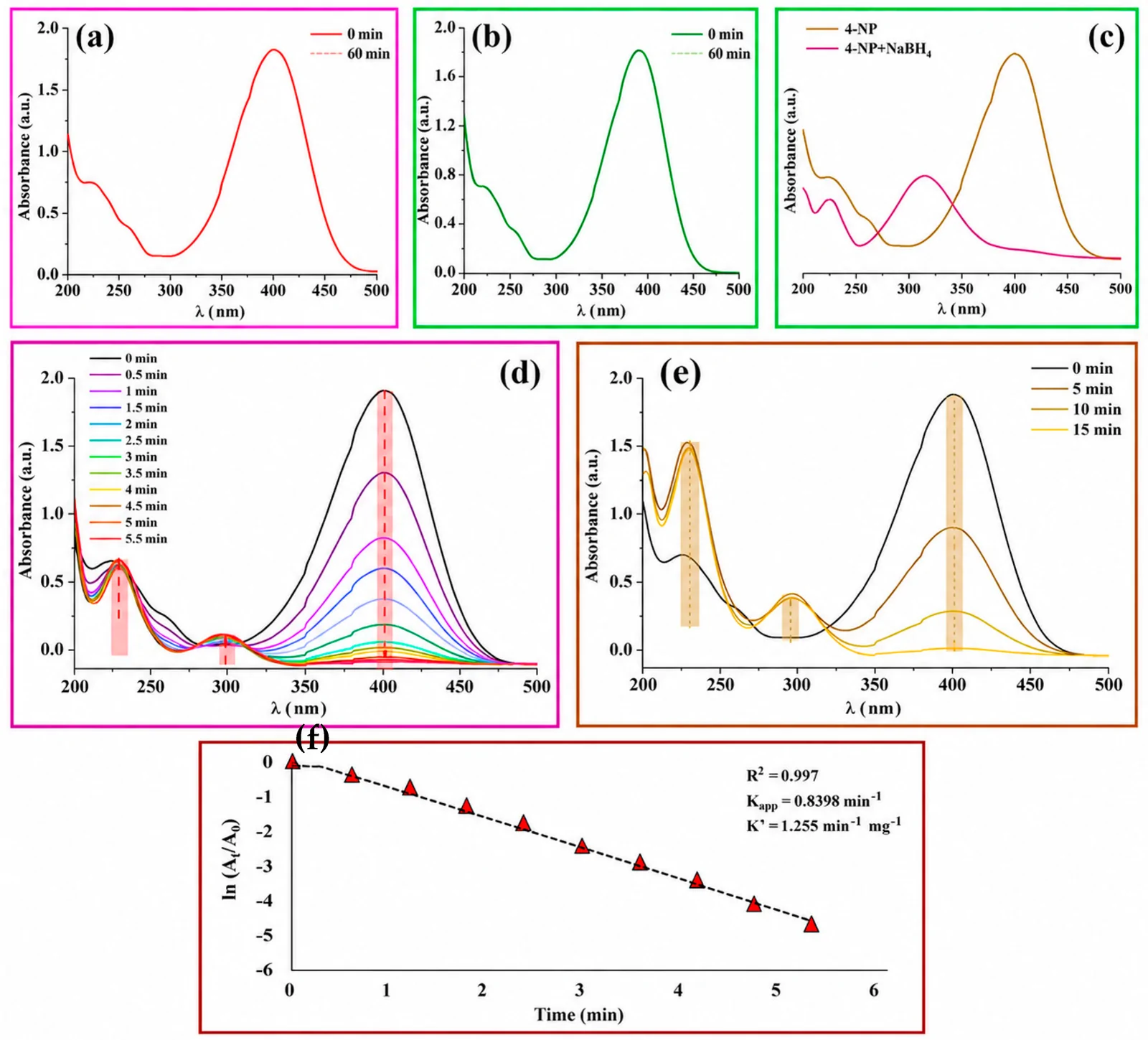
UV-Vis absorption spectra under optimal conditions of (**a**) 4-NP with NaBH_4_ in the absence of catalyst, (**b**) 4-NP with NaBH_4_ in the presence of biochar as a catalyst, (**c**) 4-NP and 4-NP with NaBH_4_, (**d**) time-dependent UV–vis spectra of 4-NP reduction in the presence of 12.5%Co_3_O_4_/biochar catalyst, (**e**) 4-NP with NaBH_4_ in the presence of Co_3_O_4_ as a catalyst, and (**f**) the plot of ln(C/C_0_) versus time.

Figure 14c illustrates an absorption peak at 317 nm for the 4-NP solution. This peak vanished when NaBH_4_ was added and shifted to 400 nm, which is ascribed to the conversion of 4-NP into 4-nitrophenolate ion [61]. The intensification of the 4-NP solution color from light yellow to dark yellow provides evidence of the formation of phenolate ions. Figure 14a depicts the absorption spectra of 4-NP using only NaBH_4_ (i.e., without a catalyst), which showed negligible catalytic performance even after 60 min. Although the standard reduction potentials for 4-NP/4-AP and H_3_BO_3_/BH_4_^−^ demonstrate that the reduction of 4-nitrophenol to 4-aminophenol in the presence of sodium borohydride is thermodynamically favorable, the reaction efficiency is inhibited by a considerable kinetic barrier resulting from the wide potential gap between the electron donor (BH_4_^−^) and the acceptor (4-NP) [58]. The catalyst acts as a reliable electron mediator that ensures charge transfer during the reduction. Similarly, the reduction of 4-NP by NaBH_4_ was performed in the presence of biochar (0% Co_3_O_4_/biochar), and no significant change in the absorption spectra of 4-NP was observed (Figure 14b). For comparison, the catalytic efficiency of Co_3_O_4_ NPs in the reduction of 4-NP by NaBH_4_ was studied (Figure 14e). The reduction reaction proceeded at a relatively slow rate, reaching completion within approximately 15 min. In contrast, in the presence of 12.57% Co_3_O_4_/biochar composite and NaBH_4_, 4-NP was rapidly converted to 4-AP, achieving a reduction of ~100% in only 5.5 min. A new peak emerged around 296 nm, which was related to the characteristic absorption bands of 4-AP, as illustrated in Figure 14d. HPLC analysis was not accessible in the present study and is therefore considered an important direction for future work.

To further assess the contribution of the interaction between Co_3_O_4_ and biochar, the reduction time was compared with that of the corresponding physical mixture prepared at the same Co_3_O_4_ loading under identical reaction conditions (Table 6). The physical mixture required 12.5 min for complete reduction, whereas the 12.57% Co_3_O_4_/biochar composite achieved complete reduction within 5.5 min. Thus, the composite exhibited a reduction time approximately 2.3 times shorter than that of the physical mixture.

This high catalytic activity and the complete reduction in the 4-NP concentration within 5.5 min over the 12.57% Co_3_O_4_/biochar catalyst can be attributed to the well-dispersed Co_3_O_4_ nanoparticles and the strong complementary contribution between Co_3_O_4_ and biochar.

The kinetic analysis was performed under the optimized reaction conditions, with NaBH_4_ maintained at a fixed concentration and used in excess in the reaction medium compared to 4-NP. Under these conditions, its concentration could be considered approximately constant throughout the reaction. Therefore, the reaction was analyzed using pseudo-first-order kinetics to calculate the rate constant K_app_ [62,63]:

(2) $$\ln\left(\frac{C_{t}}{{\;C}_{0}}\right)=\ln\left(\frac{A_{t}}{{\;A}_{0}}\right)=K_{\mathrm{app}}\times t$$

where A_t_ is the absorbance at t of the reaction and A_0_ is the initial absorbance.

In this study, no induction time was identified during the reduction process, which indicates that reactants and products diffuse in and out of the catalyst rapidly. This leads to a high reduction rate and proves the efficiency of the 12.57%Co_3_O_4_/biochar catalyst. It should be noted that induction time in the reduction reaction of organic materials using NaBH_4_ was observed in some reported studies [7].

The ln $\left(\frac{A_{t}}{\;A_{0}}\right)$ vs. the time of reaction (t), obtained by the analysis of the absorption peak intensities at 400 nm (Figure 14f), shows a linear relationship, which confirms the good fitting of 4-NP reduction kinetics to the pseudo-first-order kinetics model. The apparent rate constant (k_app_) was determined to be 0.8398 min^−1^. The activity factor, k′ $\left(\min^{-1}/\mathrm{mg}\right)$, was also calculated according to the following equation [62,63]:

(3) $$K^{\prime }=\frac{K_{\mathrm{app}}}{m}$$

where m is the mass of the cobalt oxide engaged in the 4-NP reduction.

In this context, the resulting value of k′ was 1.26 $\min^{-1}/\mathrm{mg}$.

For comparison, previous reports for the reduction of 4-NP using catalysts were presented in Table 7. As shown, the 12.5% Co_3_O_4_/biochar catalyst exhibits a significantly higher activity factor (k’ = 1.256 min^−1^mg^−1^) compared to many previously reported catalysts. This enhanced catalytic activity can arise from the key physical characteristics of the nanocomposite. Specifically, the well-developed porous structure, inherited from the pyrolyzed sludge, likely minimizes mass-transfer limitations for the 4-NP reactant. Furthermore, the homogeneous dispersion of Co_3_O_4_ nanoparticles, as confirmed by TEM analysis, ensures a high density of accessible catalytically active sites.

### 2.5. Proposed Mechanism for Catalytic Reduction of 4-NP

**Figure 15:**
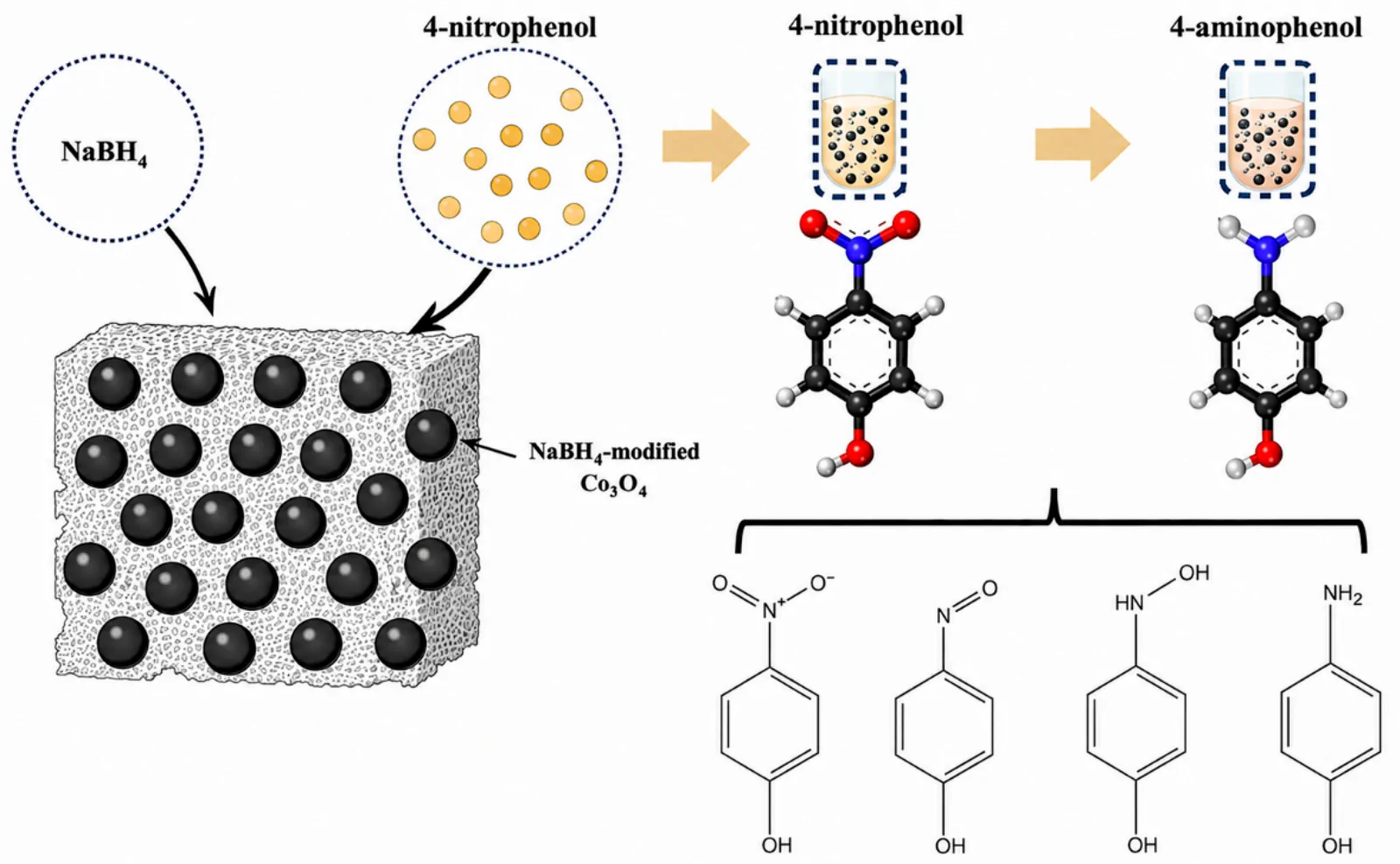
Proposed mechanism of catalytic reduction of 4-NP over the prepared Co_3_O_4_/biochar nanocomposite.

The catalytic reduction of 4-nitrophenol (4-NP) to 4-aminophenol (4-AP) can be described by either the Eley–Rideal (ER) or Langmuir–Hinshelwood (LH) models. The LH model is widely used for the heterogeneous catalytic reduction of 4-NP in the presence of NaBH_4_ [12,53]; therefore, this study focuses on the LH model for this reaction (Figure 15). In the LH mechanism, the reducing agent BH^−^_4_ and p-nitrophenol are adsorbed onto the catalyst surface, where they interact to form a product that subsequently desorbs [67]. It should be taken into consideration that, according to this mechanism, intermediates can be formed during the reaction, and the rate of the catalytic hydrogenation is highly dependent on the rate of adsorption of the reactive species onto the nanocatalysts surface [61].

Figure 15 illustrates the proposed mechanism for the reduction of 4-NP in the presence of Co_3_O_4_/biochar catalyst. The reaction process begins when NaBH_4_ is added to the solution. The dissociation of NaBH_4_ generates hydride ions (H^−^), which are a prerequisite for reducing the nitro group in 4-NP. These species are involved in the electron-transfer process during the reduction reaction. Once dissociated, 4-NP is introduced into the solution containing the Co_3_O_4_/biochar catalyst. The 4-NP molecules are attracted to the catalyst and are attached to the catalytically active surface sites of the NaBH4-modified cobalt oxide dispersed on the biochar surface. The hydride ions (H^−^) provided by NaBH_4_ are then transferred to the nitro group of 4-NP, initiating its reduction to an amine group -NH. The Co_3_O_4_/iochar catalyst is of great importance, as it provides a surface environment that facilitates the transfer of electrons from NaBH_4_ to the 4-NP molecules. The catalyst facilitates the reduction process, thereby accelerating the overall reaction. In particular, for every molecule of 4-NP, each molecule of 4-NP accepts six electrons (6e^−^) and six protons (6H^+^), which eventually transform the 4-NP into 4-aminophenol (4-AP), an industrially relevant compound. This reduction mechanism not only points out the importance of the NaBH_4_-modified cobalt oxide surface but also the biochar’s role as a support material that boosts the catalytic efficiency of the metal oxide catalyst [68,69].

The determination of the surface valence states by XPS, which was not accessible in the present study, is required to confirm the proposed pathway and constitutes the next stage of this work.

### 2.6. Reusability Study of 12.5% Co_3_O_4_/Biochar for 4-NP Reduction

**Figure 16:**
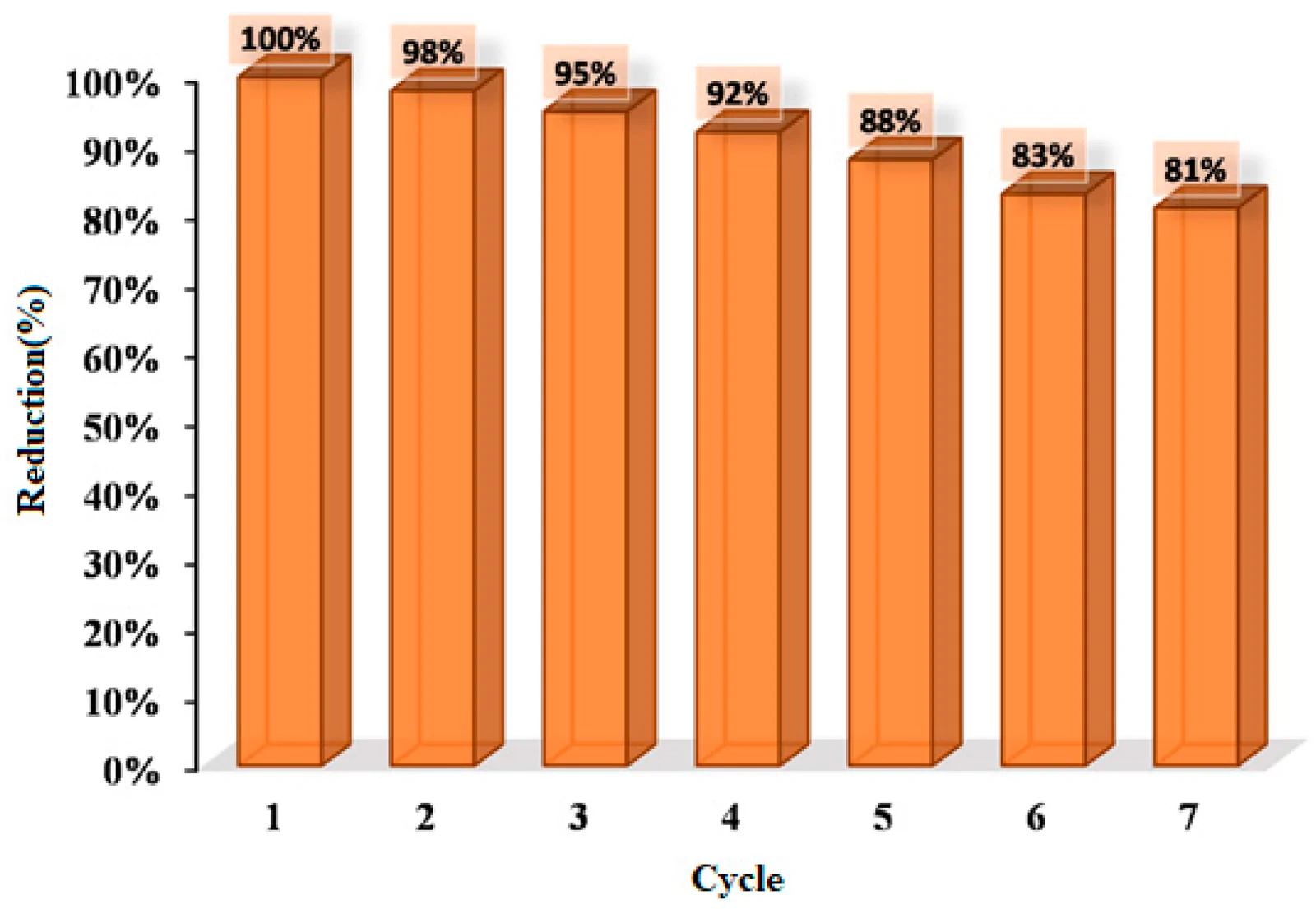
The reusability of the 12.5%Co_3_O_4_/biochar catalyst for the reduction of 4-NP.

The catalytic activity of the synthesized 12.5% Co_3_O_4_/biochar catalyst was examined over seven cycles of consecutive 4-NP reduction with NaBH_4_ as the reducing agent. Each cycle was subjected to the same evaluation by measuring the conversion efficiency with a new 5 mL of 4-NP solution. As demonstrated in Figure 16, the catalyst retained its full performance up to the fourth cycle, presenting reduction rates of 100%, 98%, 95%, and 92%, respectively. In the following cycles, a gradual decrease in efficiency was noticed, reaching 81% in the seventh cycle. The reason for this small drop is due to the combined effects of partial loss of catalyst during recovery and possible surface fouling; nevertheless, it points to the very high stability and good recyclability of the composite [58,70].

**Figure 17:**
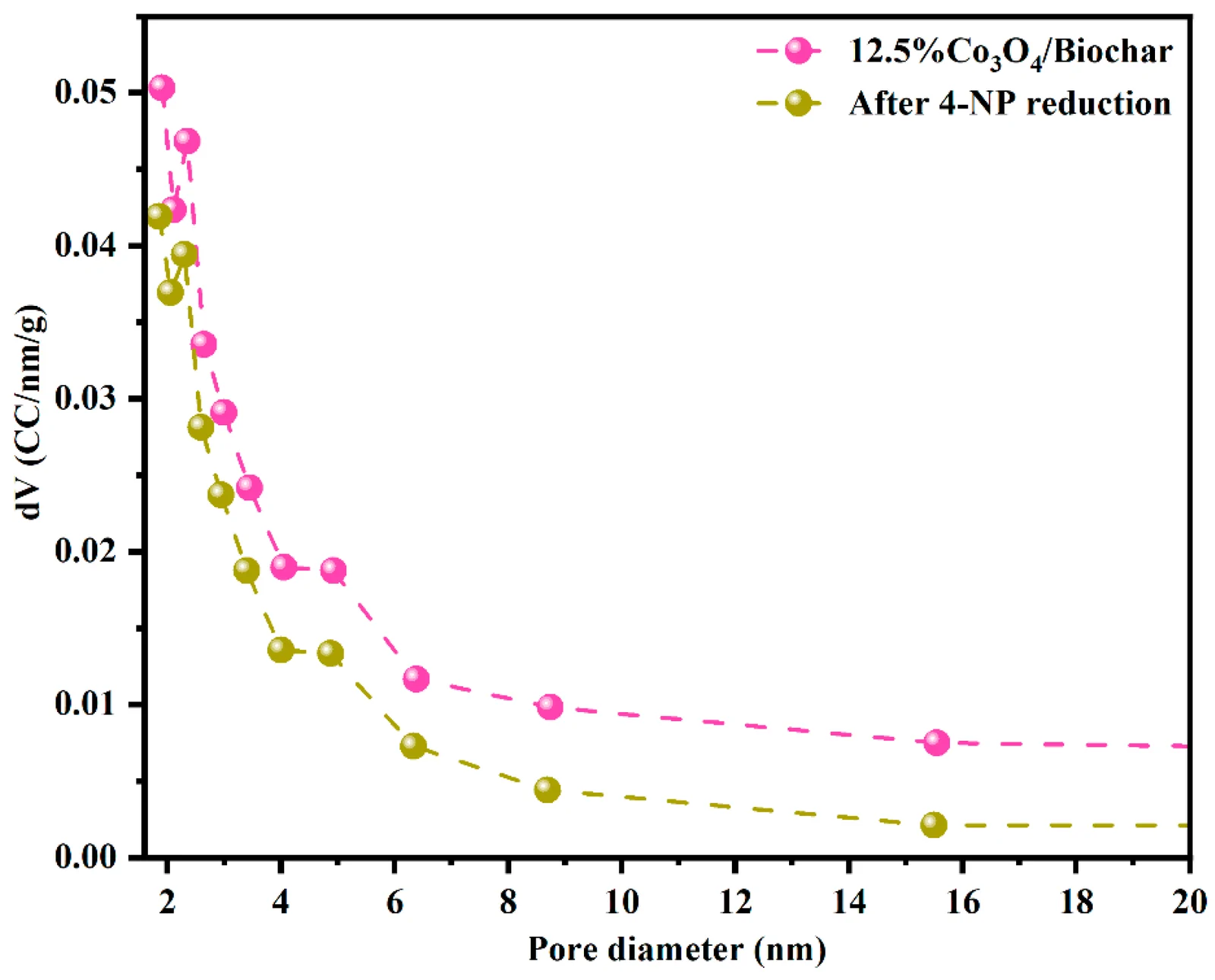
BJH pore size distribution of the 12.5% Co_3_O_4_/biochar catalyst before and after 4-NP reduction.

To further evaluate the structural stability of the catalyst after the 4-NP reduction, N_2_ adsorption–desorption analysis was performed to compare the textural properties of the 12.5% Co_3_O_4_/biochar catalyst before and after the reaction. As shown in Figure 17, the BJH pore size distributions of the catalyst before and after the reduction cycles exhibit similar profiles, with the main contribution located in the small mesopore region (~2–4 nm) and a continuous pore distribution extending toward larger pore diameters. After the 4-NP reduction, the specific surface area decreased from 75.7 to 67.3 m^2^/g, corresponding to a relatively small decrease of about 11% (Table 8). Similarly, the total pore volume decreased from 0.198 to 0.129 cm^3^/g (Table 7), while the overall pore size distribution remained qualitatively similar. The preservation of the main pore size distribution indicates that the porous carbon matrix and its accessible mesoporous structure were largely maintained after the catalytic reaction. The moderate decrease in surface area and pore volume may be attributed to partial pore blockage by adsorbed reaction species or minor structural rearrangements during the reaction, rather than a severe collapse of the porous network. Overall, the retention of approximately 90% of the initial BET surface area after the reaction demonstrates the good textural stability of the 12.5% Co_3_O_4_/biochar catalyst, which is favorable for its recyclability and sustained catalytic performance.

Therefore, the decrease in catalytic efficiency may be due to minor catalyst losses during the repeated recovery and washing procedure or to the slight changes in the textural properties observed after repeated use. Nevertheless, the catalyst retained 81% of its initial catalytic efficiency after seven consecutive cycles, which points to the good reusability of 12.5% Co_3_O_4_/biochar catalyst [58,70].

## 3. Materials and Methods

### 3.1. Materials

CoCl_2_·6H_2_O (Carlo Erba, Milan, Italy), NaOH (Sigma-Aldrich, St. Louis, MO, USA), and C_2_H_5_OH (Carlo Erba, Milan, Italy) were among the chemicals that were used in this study. LOBA Chemie (Mumbai, India) supplied the 4-nitrophenol (C_6_H_5_NO_3_) and sodium borohydride (NaBH_4_). No further purification steps were applied. The biochar (BC) production concerned the wastewater sludge that was obtained from the wastewater treatment facility located in Ifrane, Morocco. All synthesis steps were performed with distilled water.

### 3.2. Synthesis of Nanocomposite

#### 3.2.1. Preparation of Porous Biochar (BC) from Sewage Sludge (SS)

The procedure for converting sewage sludge into biochar involved multiple steps. First, the sludge was dried at 120 °C for 48 h in an oven, crushed, and sieved to produce fine particles. The obtained powder was pyrolyzed in a programmable muffle furnace under a limited oxygen atmosphere with a heating rate of 10 °C/min and at a temperature of 550 °C for 1 h. Then, 10 g of the pyrolyzed product was dissolved in 0.5 M HCl and stirred for 5 h. After that, the obtained solid was washed multiple times with distilled water until neutralization. Finally, the material was dried in an oven at 80 °C for 24 h to get the biochar.

#### 3.2.2. Synthesis of Cobalt Oxide–Biochar Composites (Co_3_O_4_/Biochar)

**Figure 18:**
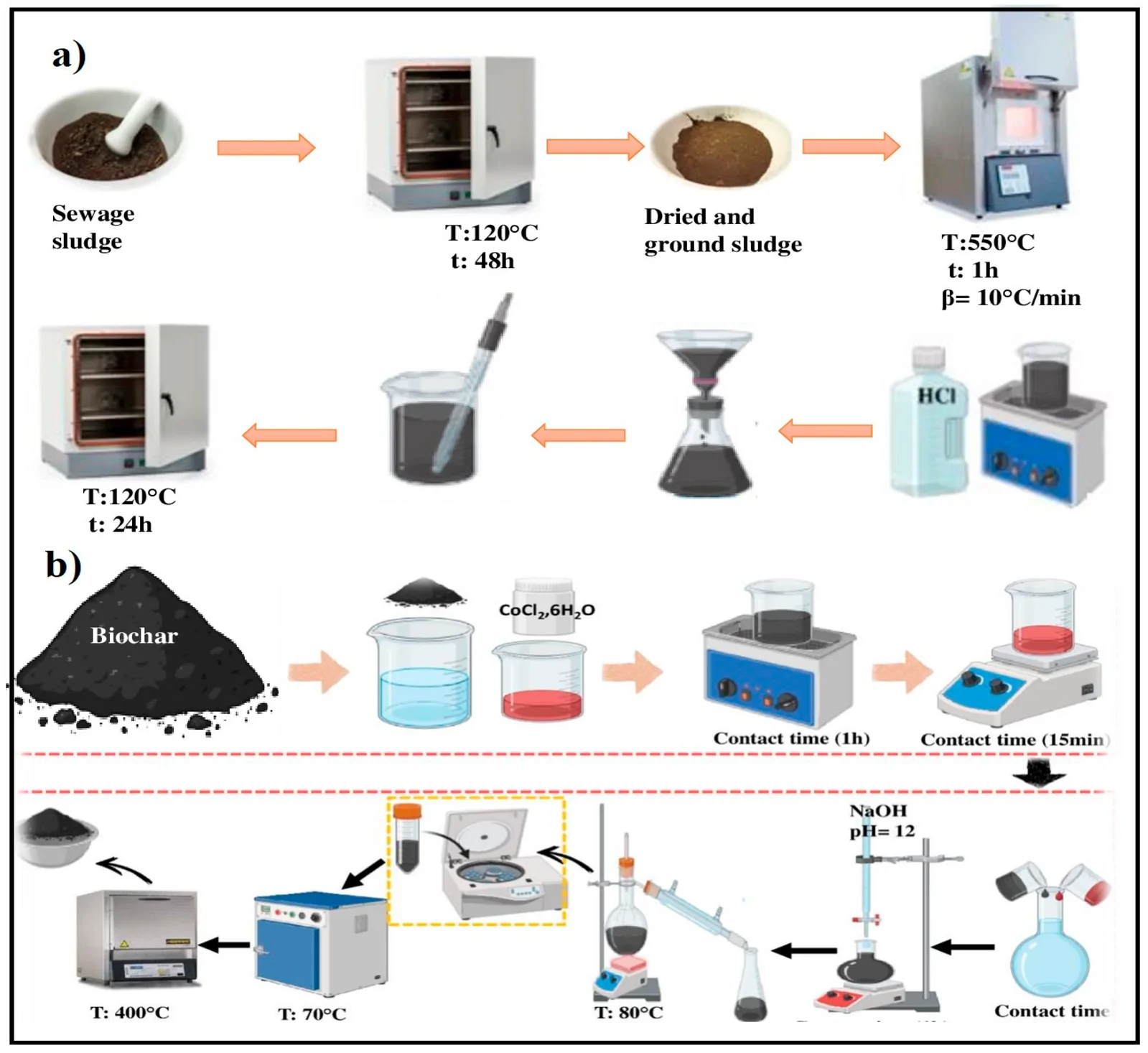
Schematic illustration of preparation of (**a**) biochar and of (**b**) Co_3_O_4_/biochar composite.

The composites were synthesized through the wet impregnation method, during which both ultrasonication and heating were applied. This technique produces a composite with a stable and uniform distribution of oxide nanoparticles on biochar support. Then, 1 g of BC powder was ultrasonicated for 1 h in 50 mL of distilled water. A predetermined quantity of CoCl_2_·6H_2_O was dissolved in 20 mL of distilled water, and the solution was stirred magnetically for 15 min. The two solutions were mixed in a flat-bottom flask for 30 min under continuous stirring. Then, NaOH was slowly added to adjust the pH of the solution to about 12. The mixture was heated to 80 °C and refluxed for 2 h while stirring constantly with a mounted reflux condenser. The precipitated product was centrifuged, then washed six times with distilled water and ethanol, and finally dried in an oven at 85 °C overnight. For generating the Co_3_O_4_/biochar composite, the material was calcined at 400 °C in air for 1 h. The different oxide loadings (wt.%) were attained by changing the concentration of the cobalt precursor while preparing the samples. The composites created were labeled as xCo_3_O_4_/biochar, with x = 2%, 5%, 10%, 12.5%, 15%, and 20%. Pure cobalt oxide was also prepared as a control sample, without the addition of BC, following the same procedure, and was referred to as Co_3_O_4_. The detailed sample preparation procedure is shown in Figure 18.

### 3.3. Surface Charge Determination: pH_pzc_ Measurement

Surface zero charge point (pH_pzc_) is the pH value corresponding to the charge neutrality of the material surface [71]. To find this value, six NaCl solutions (0.01 M, 50 mL each) were made with the initial pH values ranging from 2 to 12. Then, 20 mg of the analyzed material was added to each of the solutions, and the mixtures were stirred for 48 h at room temperature. Thereafter, the solutions were followed by filtration, and the final pH was determined [72].

The pH at zero surface charge (pH_pzc_) was obtained from the equation below:

(4) $$\Delta \mathrm{pH}={\mathrm{pH}}_{f}-{\mathrm{pH}}_{i}$$

where pH_f_ is the final pH, while pH_i_ refers to the initial pH.

### 3.4. Characterization Techniques

A set of analysis techniques was used to characterize the structural, morphological, optical, and thermal properties of the synthesized materials. X-ray diffraction (XRD) was utilized to identify the crystalline phases, and a Shimadzu 6100 diffractometer from Shimadzu Corporation, Kyoto, Japan, operating at 40 kV and 30 mA was used, with monochromatic Cu Kα radiation (λ = 0.1549 nm). The diffraction patterns were measured in a 2θ range of 10–70° with a step size of 0.02° and a scanning rate of 2°/min, and the phase was determined by comparing with the reference data from the International Centre for Diffraction Data (ICDD). The UV-Vis-NIR optical spectrometer of a PerkinElmer Lambda 900 (PerkinElmer, Waltham, MA, USA) was set up for the 200–500 nm range to examine the optical absorption properties during the real-time reduction of 4-nitrophenol (4-NP). Morphology, elemental distribution, and elemental mapping were analyzed by means of environmental scanning electron microscopy coupled with an energy-dispersive X-ray spectrometer using a Thermo Fisher microscope from Thermo Fisher Scientific, Bend, OR, USA. Fourier transform infrared spectroscopy of Jasco FT/IR 4600, from Jasco Corporation, Tokyo, Japan, was used to obtain, in the range of 4000–400 cm^−1^, the spectral data of functional groups with a resolution of 4 cm^−1^ in ATR mode. N2 adsorption–desorption isotherms were used along with the Brunauer–Emmett–Teller (BET) method to ascertain specific surface area, pore volume, and pore size distribution using a Nova Touch LX4 analyzer (Quantachrome Instruments, Boynton Beach, FL, USA). The decomposition behavior and the thermal stability of the produced powders were analyzed via TGA/DTA (thermogravimetric and differential thermal analyses) using a Labsys™ (1F) Setaram system from Setaram, Caluire-et-Cuire, France, wherein light and air-opened conditions were used to heat 10 mg of the sample from 30 °C to 800 °C at a rate of 10 °C/min. Transmission electron microscopy (TEM) was carried out on a Tecnai G2 microscope (FEI Company, Hillsboro, OR, USA) operating at 120 kV with a spatial resolution of 0.35 nm. Elemental CHNS analysis was conducted using a Flash Smart elemental analyzer (EA) from Thermo Fisher Scientific, OR, USA.

### 3.5. Catalytic Reduction of 4-NP

#### 3.5.1. Central Composite Design (CCD) for 4-NP Catalytic Reduction

CCD was applied in this work to optimize the conversion of 4-NP, in line with its increasingly common use in the analysis of catalytic reduction processes. It was one of the proper experimental design strategies for RSM, which allowed the generation of a polynomial model enabling the determination of the influence of variables and the optimal operational conditions. This method made it possible to assess the factors controlling the conversion of 4-NP in a systematic way. The experimental protocol created with this method specified a certain number (N) of trials to be performed according to the following equation [73]:

(5) $$N=2^{k}+2\times k+N_{0}$$

k denotes the factors involved, while N_0_ refers to the number of central point replicates, which are done in triplicate for the purpose of assessing the experimental error and checking the data reproducibility. The chosen factors for reduction were catalyst amount (X_1_), NaBH_4_ concentration (X_2_), and Co_3_O_4_ loading in the composite (X_3_). The reduction time (Y), the response variable, and the parameter ranges are reflected in Table 9.

These ranges were selected based on the preliminary experiments, with the aim of maintaining suitable reduction times for reliable kinetic monitoring.

The second-order polynomial response expression was employed to correlate the independent variables and is described as in Equation (6):

(6) $$Y=b_{0}+\sum_{i=1}^{n}b_{i}X_{i}+(\sum_{i=j}^{n}b_{\mathrm{ij}}X_{i}{)}^{2}+\sum_{J=i+1}^{n-1}\sum_{j=i+1}^{n}b_{\mathrm{ij}}X_{i}X_{j}+\varepsilon$$

The arrangement of the equation implies that Y is the dependent variable and that X_i_ and X_j_ are the independent variables. The parameters b_0_, b_i_, b_ii_, and b_ij_ represent the intercept, linear coefficients, quadratic terms, and interaction terms, respectively. The part ε denotes the residual error. The R^2^ value and analysis of variance (ANOVA) determined the reliability and statistical significance of the suggested model. Design-Expert software (13.0.5.0 version) was used for the experimental design, predictive modeling, and optimization phases.

#### 3.5.2. The Evaluation of Catalytic Efficiency of the Prepared Catalysts

The catalytic efficiency of the Co_3_O_4_/biochar catalysts were tested by the catalytic conversion of 4-nitrophenol (4-NP) to 4-aminophenol (4-AP) at room temperature with NaBH_4_ excess. In a typical procedure, 6 mg of 20% Co_3_O_4_/Biochar was dispersed in 35 mL of deionized H_2_O solution. After that, 10 mL of a 70 mM NaBH_4_ solution was added, and the mixture was sonicated for 10 min to get a good dispersion of the components. Then, 5 mL of 4-NP (3 mM) was introduced to the solution. As a result, the final concentrations in the reaction mixture were 0.3 mM for 4-NP and 21 mM for NaBH_4_. The reduction reaction was tracked by time-dependent UV–vis spectra, within the range of 200–500 nm of the spectrophotometer. The reduction efficiency of 4-NP was then calculated using the formula below [74]:

(7) $$\%\mathrm{Reduction}=\frac{\left(C_{0}\;-C_{t}\right)}{{\;C}_{0}}\times 100=\frac{\left(A_{0}\;-A_{t}\right)}{{\;A}_{0}}\times 100$$

where A_t_ and C_t_ are the absorbance and concentration of 4-nitrophenol, respectively, at time “t” of the reaction, while A_0_ and C_0_ are the initial absorbance and initial concentration.

A series of experiments was performed by systematically varying the reduction parameters (X_1_, X_2_, and X_3_) within the defined ranges. Different parameter combinations were assessed in accordance with the experimental design, while the 4-NP initial concentration was kept constant at 3 mM throughout the experiments. From the experimental data, the predictive model was constructed to identify and optimize the main parameters controlling the reduction of 4-NP. The optimal conditions were then determined, and the kinetic study of the reaction was carried out to evaluate the rate of reaction as well as to elucidate the mechanism of the reduction process. To establish the crucial role of Co_3_O_4_/biochar catalyst, a control experiment was carried out in which 4-NP reduction with NaBH_4_ was attempted, but without a catalyst. The results obtained quite clearly indicate that the significant improvement in 4-NP reduction is due to the catalytic action of Co_3_O_4_/biochar nanocomposites. Moreover, two catalytic experiments consisting of biochar only and Co_3_O_4_ nanoparticles were conducted under the optimal conditions. These experiments give a comparative evaluation of the catalytic performance of the Co_3_O_4_/biochar nanocomposites, accentuating their excellent performance toward reducing 4-NP.

The reusability and stability of the Co_3_O_4_/biochar catalyst were also examined. Successive reaction cycles were performed to determine the maximum activity loss over several uses, which would reveal the practical use and stability of the prepared catalyst. After completion of each cycle, the catalyst was separated from the reaction mixture using filtration, rinsed with deionized water and ethanol, and then dried at 80 °C for 8 h. The recovered catalyst was afterward reused under the same experimental conditions for the next cycle.

**Figure 19:**
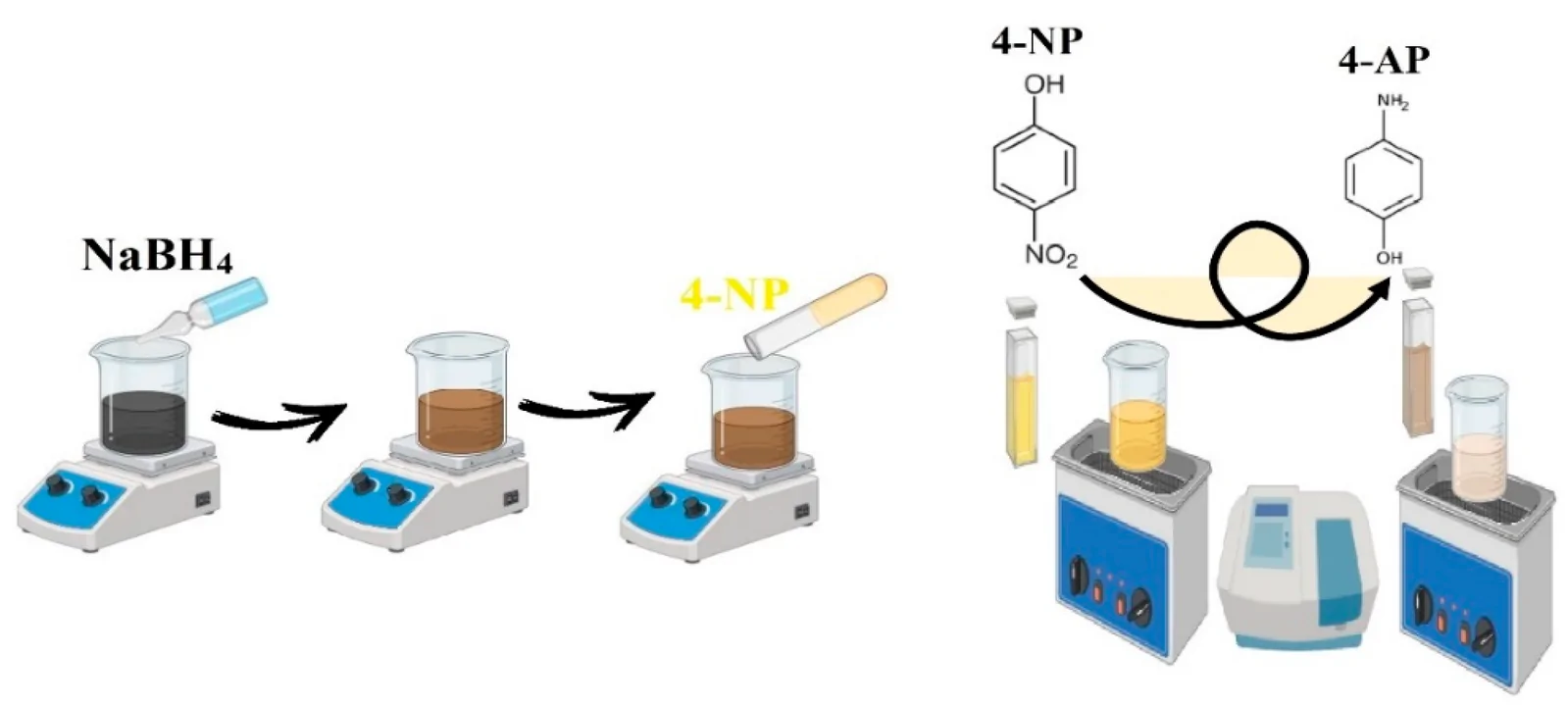
Schematic of the catalytic reduction of 4-NP experiment.

The experimental setup of the reduction process is shown in Figure 19.

## 4. Conclusions

In conclusion, a simple method was employed to obtain Co_3_O_4_ nanoparticles on the biochar made from sewage sludge. The catalysts were characterized using various methods, including XRD, ATR-FTIR spectroscopy, SEM-EDX, TGA-DTA, pH_pzc_, BET, and CNHS analysis. The Co_3_O_4_/Biochar composites exhibited remarkable catalytic activity for the reduction of 4-NP using NaBH_4_ as the reducing agent, in contrast to the moderate activity of pure Co_3_O_4_ and the negligible catalytic performance of biochar. The process of 4-NP reduction by NaBH_4_ over the Co_3_O_4_/biochar catalyst is controlled by three principal factors: dosage of catalyst, concentration of reducing agent, and amount of oxide on the composite. Under a CCD, an RSM model was developed, and the operating conditions were optimized within the investigated experimental domain. The results reveal that the established model is significant and successfully describes the experimental data. The optimal operating conditions were found to be as follows: 5.32 mg of the composite catalyst, a NaBH_4_ concentration of 26 mM, and a 13% Co_3_O_4_ loading on the composite. The RSM-based desirability optimization predicted these conditions as the optimal combination within the investigated domain. Under these conditions, the reduction of 4-NP was completed, and the reaction kinetics were monitored in about 5 min. The rate of the reduction reaction followed a pseudo-first-order model and resulted in a very high per mass active factor of 1.256 min^−1^mg^−1^. Moreover, the catalyst showed good stability and maintained its high catalytic effectiveness even after seven cycles of reuse. Overall, the results demonstrate that the Co_3_O_4_/biochar composite is an effective and reusable catalyst for the reduction of 4-NP by NaBH_4_.

## Figures and Tables

**Table 1:** Textural properties of the sewage sludge, biochar, Co_3_O_4_, and Co_3_O_4_/biochar.

Sample	SSA (m^2^/g)	V (cm^3^/g)
Sewage sludge	3.5	0.004
Biochar	50.4	0.084
Co_3_O_4_	22.2	0.012
12.5% Co_3_O_4_/biochar	75.7	0.198

**Table 2:** CHNS elemental composition (%) of sewage sludge (SS), biochar (BC), and Co_3_O_4_/biochar.

Materials	N (%)	C (%)	H (%)	S (%)
SS	1.11	33.82	1.03	0.77
BC	1.68	42.05	6.07	0.13
12.5%Co_3_O_4_/Biochar	2.04	29.84	3.52	0.85

**Table 3:** Central composite design experimental matrix for RSM optimization.

Run	X_1_	X_2_	X_3_	Y
1	6	21	12.5	5.5
2	6	30	12.5	4.25
3	10	21	12.5	3.2
4	6	21	12.5	6.5
5	10	30	20	3
6	2	30	5	15
7	2	12	5	21
8	10	12	5	12.5
9	6	21	12.5	6.75
10	2	30	20	7
11	6	21	20	4.5
12	10	30	5	8.25
13	2	21	12.5	10
14	6	21	12.5	6
15	6	21	5	9.5
16	6	12	12.5	8
17	6	21	12.5	5
18	2	12	20	12
19	10	12	20	6.75

**Table 4:** Statistical analysis of process parameters using ANOVA.

Source	Sum of Squares	Df	Mean Square	*F*-Value	*p*-Value	
Model	352.51	9	39.17	46.13	<0.0001	significant
X_1_	97.97	1	97.97	115.37	<0.0001	
X_2_	51.76	1	51.76	60.95	<0.0001	
X_3_	108.90	1	108.90	128.24	<0.0001	
X_1_X_2_	1.13	1	1.13	1.32	0.2794	
X_1_X_3_	4.50	1	4.50	5.30	0.0468	
X_2_X_3_	0.2813	1	0.2813	0.3312	0.5791	
X_1_^2^	8.01	1	8.01	9.44	0.0133	
X_2_^2^	4.18	1	4.18	4.93	0.0536	
X_3_^2^	12.19	1	12.19	14.36	0.0043	
Residual	7.64	9	0.8492			
Lack of Fit	5.59	5	1.12	2.18	0.2348	not significant
Pure Error	2.05	4	0.5125			
Cor Total	360.16	18				

**Table 5:** Statistical evaluation of model significance.

Statistical Parameters	Value
Std. Dev.	0.9215
Mean	8.14
C.V. %	11.32
R^2^	0.9788
Adjusted R^2^	0.9576
Predicted R^2^	0.9044
Adeq Precision	26.0424

**Table 6:** Comparison of 4-NP reduction performance of Co_3_O_4_/biochar-based catalysts.

Catalyst	Reduction Time (min)
Biochar	No reduction after several hours
Co_3_O_4_	15
Physical mixture of Co_3_O_4_ and biochar	12.5
12.57% Co_3_O_4_/biochar composite	5.5

**Table 7:** Comparison of the activity factor of the prepared nanocomposite with previous studies.

Entry	Catalysts	4-NP (mM)	NaBH_4_ (mM)	k_app_ (min^−1^)	k′ (min^−1^ mg^−1^)	References
1	12.5%Co_3_O_4_/Biochar	0.3	25.8	0.840	1.256	This work
2	13.6%Co@BN	1	205	0.200	0.020	[64]
3	Ag_0.008_/Co_3_O_4_	0.108	7	0.156	0.015	[13]
5	Co_3_O_4_/CoP (35%)	0.1	0.102	1.610	0.537	[65]
7	5.5Cu%/Biochar	2	80	0.270	0.066	[45]
8	RGNCO_4_	0.7	100	0.713	0.356	[66]

**Table 8:** Textural properties of the 12.5% Co_3_O_4_/biochar catalyst before and after 4-NP reduction.

Sample	SSA (m^2^/g)	Pore Volume V (cm^3^/g)
12.5% Co_3_O_4_/Biochar	75.7	0.198
After 4NP reduction	67.3	0.129

**Table 9:** Input and output variables of the reduction reaction.

**Experimental Parameters**	**Experimental Inputs**				
	**Original Variables**	**Unit**	**Quantified Levels of Coded Factors**		
			**Minimum Level (−1)**	**Central Level (0)**	**Maximum Level (+1)**
	X_1_ = catalyst dosage	mg	2	6	10
	X_2_ = concentration of NaBH_4_	mM	12	21	30
	X_3_ = Co_3_O_4_ loading in the composite	wt.%	5%	12.5%	20%
**Output variable**	Response				
	Y = Reduction time (min)				

## Data Availability

The data presented in this study are available on request from the corresponding authors.
